# Presentation matters: Impact of association of amphiphilic LPS with serum carrier proteins on innate immune signaling

**DOI:** 10.1371/journal.pone.0198531

**Published:** 2018-06-14

**Authors:** Loreen R. Stromberg, Heather M. Mendez, Jessica Z. Kubicek-Sutherland, Steven W. Graves, Nicolas W. Hengartner, Harshini Mukundan

**Affiliations:** 1 Center for Biomedical Engineering, University of New Mexico, Albuquerque, New Mexico, United States of America; 2 Physical Chemistry and Applied Spectroscopy, Los Alamos National Laboratory, Los Alamos, New Mexico, United States of America; 3 The New Mexico Consortium, Los Alamos, New Mexico, United States of America; 4 Theoretical Biology and Biophysics, Los Alamos National Laboratory, Los Alamos, New Mexico, United States of America; Science and Technology Facilities Council, UNITED KINGDOM

## Abstract

Recognition of Pathogen-associated Molecular Patterns (PAMPs) by Toll-like receptors is central to innate immunity. Many bacterial PAMPs such as lipopolysaccharide (LPS) and lipoteichoic acid have amphiphilic properties. The hydrophobicity of amphiphilic PAMPs contributes to increasing entropy and causes these molecules to self-aggregate or bind host carrier proteins in aqueous physiological environments. The goal of this work was to determine how innate immune signaling is impacted by physical presentation and association of amphiphilic PAMPs with serum carrier proteins, using LPS as an example molecule. Specifically, we measured LPS-induced cytokine profiles in murine macrophages when the antigen was presented associated with the various serum carrier proteins in serum versus a serum-depleted system. Our study demonstrates that the observed cytokine profiles are dramatically different when LPS is presented in buffer, versus in serum when it is associated with proteins, specifically with respect to inhibition of pro-inflammatory cytokines in the latter. These studies suggest that LPS-mediated cytokine expression is dependent on its presentation in physiological systems. The amphiphilicity of bacterial PAMPs and consequent association with lipoproteins is a feature, which should be taken into account in the design of *in vitro* experiments. Further studies of the interdependencies of different serum carriers can identify pathways for drug delivery and diagnostics.

## Introduction

Pattern and damage associated molecular patterns (PAMPs and DAMPs) are important signaling molecules released by bacteria, viruses, and fungi during host infection. [1, 2] Many bacterial PAMPs, such as lipopolysaccharide (LPS), [3, 4] lipoteichoic acid (LTA), [5, 6] and lipoarabinomannan (LAM) [7, 8] have evolutionarily conserved amphiphilic structures that bind innate immune receptors (e.g., Toll-like receptors) in the host to activate early immune responses. [9] Amphiphiles, such as these, can either self-aggregate or associate with carrier moieties in aqueous blood. This association is driven by the biochemical properties of the amphiphile in question, and plays a significant role in innate immune signaling and clearance. Thus, as association of amphiphiles with carriers changes *in vivo* due to a variety of physiological and environmental factors and dose concentration, so too does response to inflammation.

LPS is the predominant PAMP released by Gram-negative bacterial pathogens, which stimulates innate immunity through activation of its primary receptor complex, Toll-like receptor 4 (TLR4). [10, 11] Structurally, LPS has three primary components: the hydrophobic lipid A (also called endotoxin), and the hydrophilic core polysaccharide and O-polysaccharide antigens. [12, 13] At discrete concentrations, the amphiphilic nature of LPS induces micelle formation in aqueous matrices, due to sequestration of the hydrophobic lipid A component away from hydrophilic media (aqueous phase), to lower molecular free energy. [14] Therefore, as concentrations increase above the critical micelle concentration (CMC), LPS exists as an equilibrium of monomers and micelles. [15, 16] Many researchers have documented association of LPS with membranes of eukaryotic cells, [17] liposomes, [18] lipid bilayers, [19] giant unilamellar vesicles, [20] antibiotics, [21] serum carrier proteins, [22] chylomicrons, [23] and a host of other molecules which specifically bind LPS components. [24, 25] Thus, the biological effects of LPS are dependent, in part, on its amphiphilicity.

LPS is specifically known to associate with a variety of host serum carrier proteins, including but not limited to high-, low-, and very low- density lipoproteins (HDL, LDL, VLDL) as well as Lipopolysaccharide Binding Protein (LBP). [26, 27] HDL and LDL possess a core structure composed of cholesterol and triacylglycerides [28, 29] that associates with the hydrophobic portion (e.g., lipid A) of LPS. [23] Often, LPS micelles are transferred as monomers to serum binding proteins to facilitate clearance through the liver, [30] or to transfer LPS to receptors on immune cells. [31, 32] Therefore, these carrier proteins function as a “biological taxi service” for transporting amphipathic molecules in aqueous blood, and play a critical role in mediating the protective or inflammatory response of LPS on host cells. [23, 33] Lipoproteins have been shown to mediate detoxification of LPS-induced endotoxicity in physiological concentrations in both *in vitro* and *in vivo* studies. [34–36] Association of LPS with HDL, LDL, VLDL, chylomicrons, and other lipoproteins has been shown to reduce the lethal effect of endotoxin in rats and mice. [23, 37] LPS also associates with LBP, which plays a significant role in its presentation to the LPS-receptor, TLR4, [38] and subsequent activation of innate immunity. [39] For example, comparison of infected LBP-knockout mice and wild-type animals showed enhanced mortality and spread of bacteria in the former, suggesting that LBP plays an important role in LPS-induced inflammation. [40] However, LBP also facilitates transfer of LPS to lipoprotein carriers, which is fundamental for the neutralization of LPS and other amphiphiles (e.g., LTA). [41, 42] Thus, the balance in interactions of lipoproteins with carriers is critical to immune signaling, inflammatory response, and virulence. Yet, the interactions of LPS with its carriers in the host are often ignored in the design of *in vitro* studies, wherein LPS is presented to cells in aqueous buffers, rather than host-carrier assemblies.

Upon activation of TLR4, LPS can cause an array of events resulting in the release of cytokines and chemokines, which mediate the host inflammatory response. [43] However, reports on LPS-mediated induction of cytokines and chemokines are often conflicting, [44] which may be a consequence of differential interactions of LPS with carrier proteins, thereby making it difficult to interpret the biological significance of these findings. [45] Thus, a physiologically relevant *in vitro* system that accurately represents the LPS-induced cytokine expression, as it occurs in the infected host, does not currently exist. Here, we have explored the impact of LPS presentation on TLR4-dependent cytokine production in a murine cell system. Specifically, we have investigated whether the association of LPS with serum lipoproteins or LBP alters the pro-inflammatory cytokine profile, as compared to LPS in a system devoid of serum carrier proteins. The studies herein are not designed to reflect the cytokine profiles associated with LPS induction in the host, or advocate a single physiologically relevant approach for studying cytokine expression *in vitro*, but rather aim to highlight the impact of presentation of LPS and its association with host carriers on the observed inflammatory signatures. Our findings shed light on the importance of the presentation method of LPS for cell signaling, and the need for its understanding and incorporation in experimental design for the effective development of diagnostics and drug-delivery systems, as well as improved understanding of host-pathogen biology.

## Results and discussion

**Fig 1:**
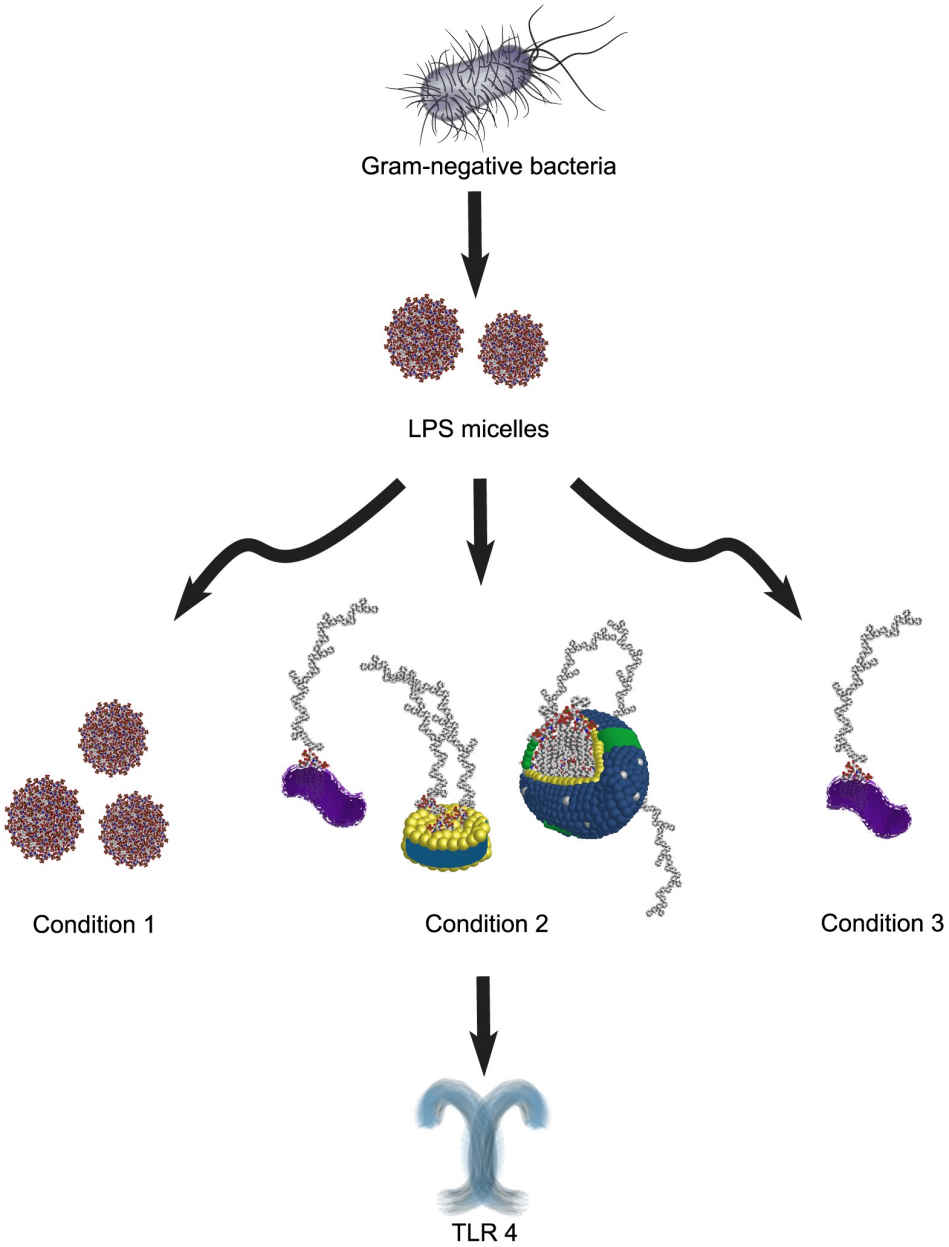
Schematic of the conditions of LPS presentation in media. **Condition 1** is when LPS is presented to TLR4(+) cells in serum-free media (buffer), though below the CMC, LPS can also present as a monomer. **Condition 2** demonstrates association of LPS with some of the common carrier proteins in serum; LBP, HDL, and LDL. **Condition 3** shows presentation of LPS to cells with de-lipidated serum, when serum carrier proteins HDL and LDL are inactivated by de-lipidation, but LBP functionality remains.

We studied the difference in cytokine and chemokine signaling of murine macrophages, when LPS was presented under three different conditions (Fig 1): condition 1—antigen presented in media (serum-free), condition 2—antigen presented in 50% murine serum supplemented culture media, and condition 3—antigen presented in 50% de-lipidated murine serum and media. These three experimental conditions will henceforth be referred to as 1, 2, and 3, respectively. For the effective design and interpretation of these studies, the CMC of LPS in buffer (PBS) was measured using a fluorescence probe method and was found to be between 1.25 and 2.5 μg/mL (S1 Fig). The CMC of amphiphiles can be impacted by numerous factors (i.e., temperature, proteins, ionic strength, and surfactants), which effects the role of amphiphilic PAMPs in several biological processes. [46–48] The impact of serum on the CMC of LPS and other amphiphilic PAMPs is a critical question requiring further investigation, which is necessary for the effective design of cell studies. For the purpose of this manuscript, the CMC measurements confirm that the concentration of LPS present in buffer are below the CMC. As noted elsewhere in the manuscript, LPS associates with carrier lipoproteins, binding proteins and other molecules in complex matrices, such as serum. These associations impact its ability to form micelles, and consequently, the CMC of the molecule in these conditions. This difference impacts the outcome of *in vitro* experiments depending on the cell media and supplements used, which was the primary purpose of our study. Thus, while the CMC of LPS in buffer may be used as an indicator of its endotoxic activity, it is important to note that this effect manifests differently in physiological systems, and may be one of the contributing factors for the outcomes of the measurements in our study.

The purpose of these measurements is not to ascertain the optimal method for use of LPS for *in vitro* studies, but to determine the existence of inherent differences in signaling patterns based on varied presentation of the amphiphile. Cytokine immunoassays kits used in previous studies have identified several limitations, including poor performance for specific cytokines and uncertainty associated with predicted outcomes. [49] To help overcome discrepancies seen in previous studies, we have designed and implemented controls to reduce inter-assay variability, which will be consistently evident throughout the various groups being presented herein.

**Fig 2:**
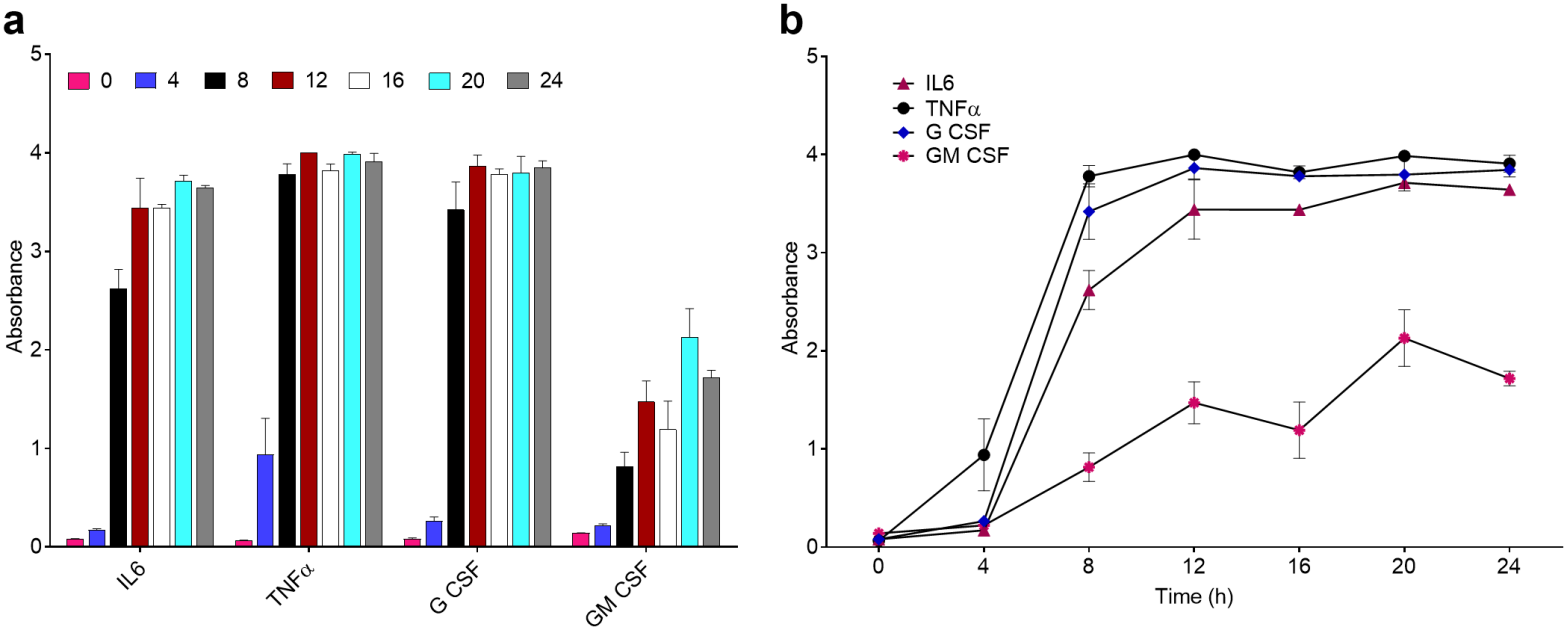
Absorbance measurements from time point study of TLR4+ cells using condition 1. **(a)** Bar graph representation of cytokines measured over a 24-hour period. The time point 0 h indicates a (–LPS) control to indicate the response of the cells after 24 h with no antigen exposure. **(b)** Selected TLR4 relevant cytokines plotted over time to demonstrate temporal response. Values are plotted as the mean absorbance (n = 3) with error bars indicating standard deviation of the mean.

The first step in these studies was to determine the optimal exposure time to effectively measure differences in cytokine expression under these different conditions. For this, we examined LPS-dependent expression of 12 cytokines in a TLR4 positive (TLR4(+)), IC-21, murine macrophage cell line at 4-hour intervals, over a 24-hour period using condition 1. While there is significant disparity in the reported literature, [32, 50] the cytokines commonly associated with TLR4 dependent signaling include, but are not limited to, interleukin-1 alpha (IL-1α), interleukin-1 beta (IL-1β), interleukin-6 (IL-6), tumor necrosis factor alpha (TNFα), interferon gamma (IFNγ), along with others such as interleukin-12 (IL-12), granulocyte (macrophage) colony stimulating factors (G CSF and GM CSF), and macrophage inflammatory proteins (MIPs). [51, 52] Fig 2a demonstrates the expression of 4 of these cytokines in TLR4(+) cells at different time points (0, 4, 8, 12, 16, 20, and 24 hours), measured using an absorbance assay, in the presence of 100 ng/mL LPS in serum-free media (condition 1). The time point zero hours (–LPS) is used as a negative control, as it monitors the cytokine activity of the cells in the absence of LPS and serum over the duration of the experiment, and indicates that no significant cytokine activity was recorded as a result of culturing or washing of cell lines. The time-dependent profiles of the primary TLR4 associated cytokines implicated with LPS-mediated induction of TLR4 is indicated in Fig 2b. The additional 8 cytokines that were measured using this assay are presented in S2 Fig. To help identify dose and time dependent response of the cells to LPS, as well as to validate our hypothesis of using different media conditions to deliver LPS, we elected to use a 12-plex absorbance-based kit to measure cytokine expression due to ease of use and cost effectiveness of the kits. The absorbance-based measurements were performed for the 12 cytokines included in the ELISArray kit, and raw data is reported in S1 Appendix and S2 Fig. Our results demonstrate peak expression of several cytokines after 8 hours in condition 1 (Fig 2a), beyond which, we observed saturation, which was not conducive for the proposed comparative analysis. We observed significant increases in IL-6, and TNFα, as well as G CSF and GM CSF, as compared to the negative kit control (S2 Fig) and (–LPS) cell control (Fig 2a, 0 h). However, we did not observe an increase in expression of IL-1β or IFNγ, as previously reported in the literature. [53] It has been shown that LPS induction of IL-1β, is specific to certain cell types, which may explain this lack of signal. [54] In addition to the expected cytokines, G CSF and GM CSF are both glycoproteins released by macrophages in response to inflammation, and are commonly associated with increased cell survival. [55] These results are consistent between replicates (n = 3) for the cell lines used here, and 8 hours of exposure was selected for subsequent experiments based on these findings.

**Fig 3:**
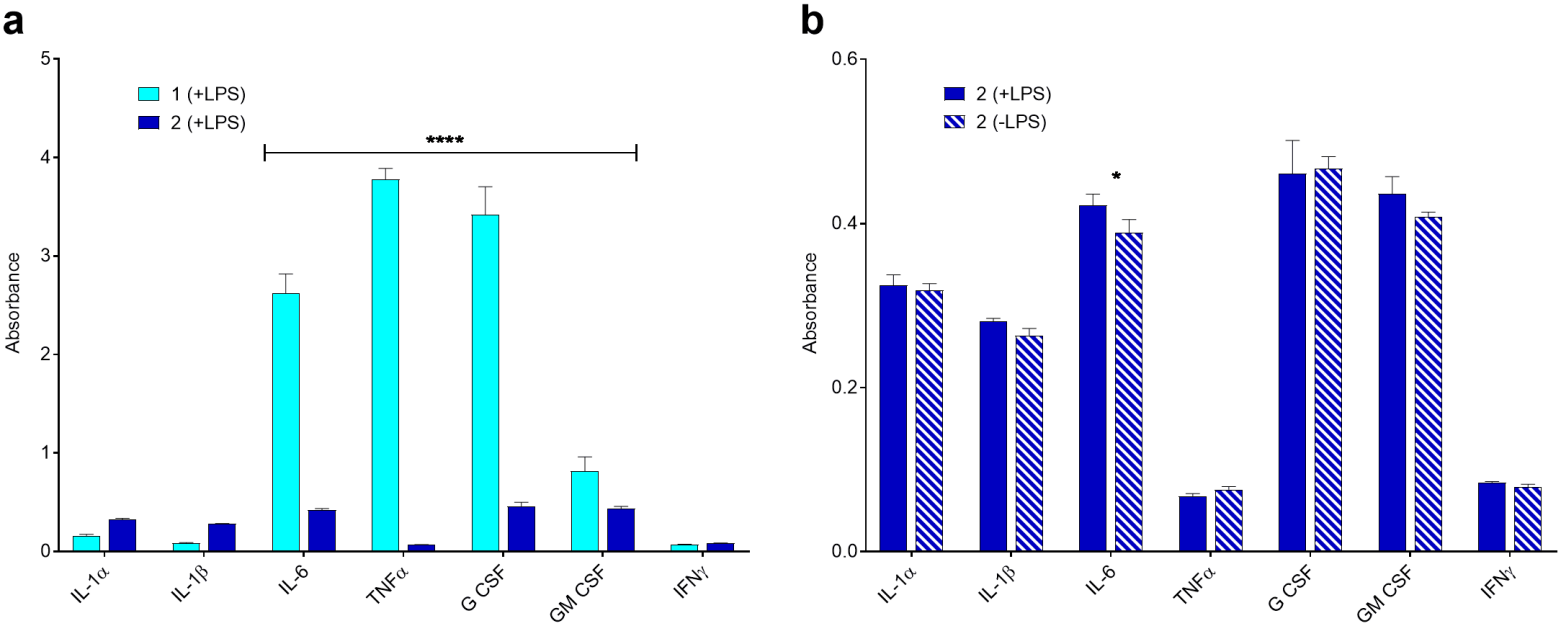
Cytokine response of cells when LPS is pre-incubated overnight with serum. **(a)** Absorbance measurements comparing condition 1 to condition 2 with LPS. **(b)** Condition 2 cytokine response of cells in the presence and absence of LPS. Values are plotted as the mean absorbance (n = 3) with error bars representing the standard deviations. Analyzed using 2-way ANOVA and Sidak’s multiple comparisons test with α = 0.05. **** p<0.0001, * p<0.05, data points lacking asterisks are considered not significant. All cytokines within a bracket carry that p-value between condition 1 and condition 2 for individual cytokines (i.e., IL-6 has level of significance between 1 (+LPS) and 2 (+LPS)) and is not meant to compare the difference between all cytokines. Statistical analysis is available in S1 Appendix.

To determine the effect of LPS on cells using condition 2, the antigen stock (5 mg/mL) was sonicated vigorously using a bath sonicator (see Methods), then mixed with serum and allowed to incubate for 24 h prior to treating cell cultures. Sonication minimizes variability in LPS micelle size (at concentrations > CMC) to ensure a homogenous mixture before mixing with serum, and the extended incubation time facilitated maximal association of LPS with the serum carrier proteins (Fig 1, condition 2) [33]. When comparing the two, it is evident that condition 1 (LPS in serum-free media) elicits a much greater cytokine induction (3x increase with select cytokines) compared to condition 2 (Fig 3a), as measured by absorbance. In fact, the cytokine response of cells when LPS was pre-incubated with serum (condition 2) was comparable to control cell populations that received no LPS treatment (Fig 3b). While exposure to murine serum may elicit a minor cytokine response, the levels of cytokines measured in the condition 2 (–LPS) control was equivalent in most cases to the negative kit control of assay buffer (S2 Fig). However, we did notice mildly elevated levels of G CSF in serum, as compared to condition 1 (–LPS) and the negative kit control, which could be explained by the potential presence of trace concentrations of either PAMPs or cytokines in the serum (S2 and S3 Figs). This data (Fig 3) demonstrates that the secretion of pro-inflammatory cytokines is markedly reduced when LPS is presented in association with serum carrier proteins, suggesting a strong protective effect of serum on LPS-induced cytokine production. These findings have significant implications not only for developing effective *in vitro* models for LPS, but also for other amphiphilic PAMPs that have similar structures, such as LTA and LAM. [56]

**Fig 4:**
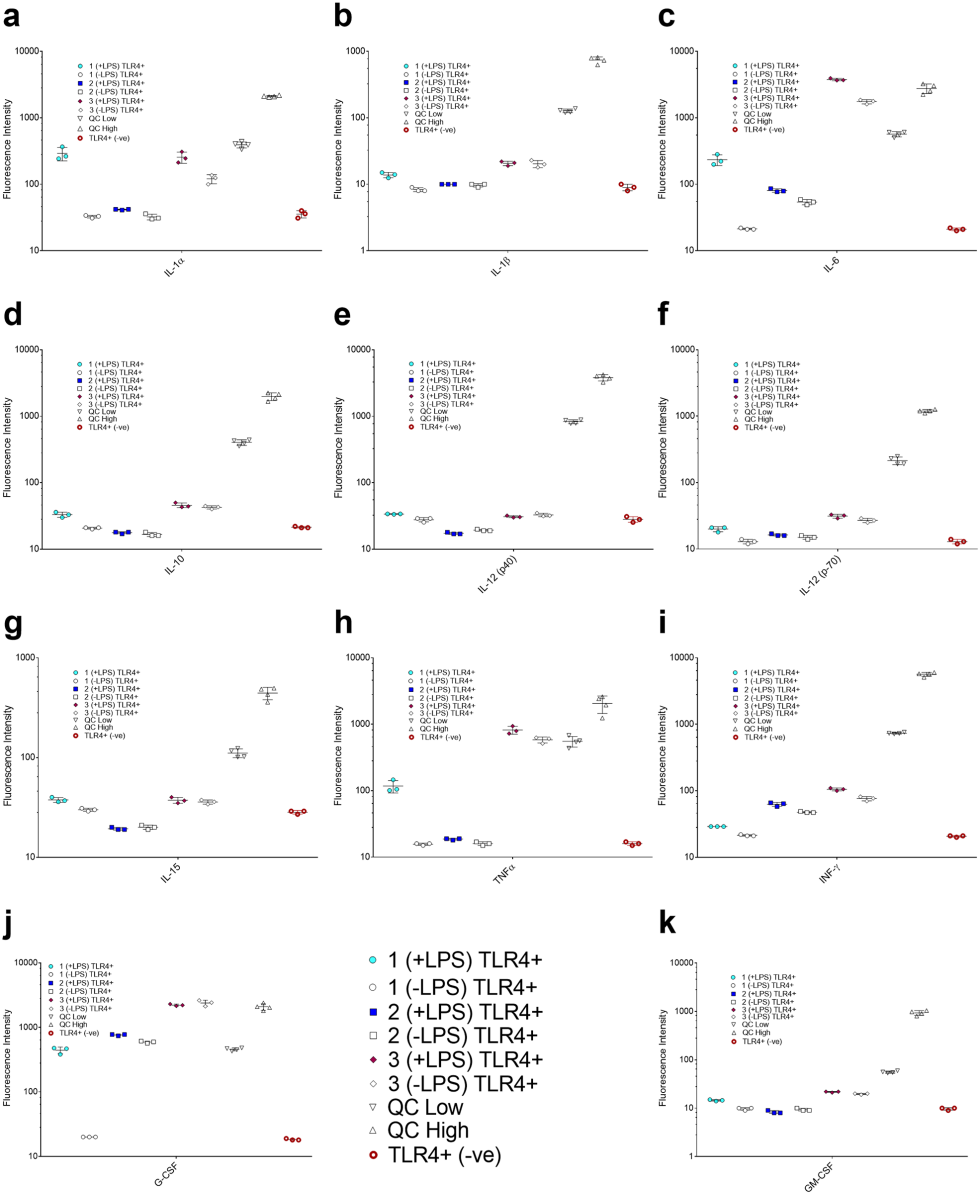
Comparative cytokine expression between conditions 1, 2 and 3 in TLR4(+) cells. Cluster plot of results of three independent replicates, plotted as fluorescence intensity for each individual cytokine, with error bars indicating the standard deviation of the mean for each condition. QC High and QC Low were high and low quality controls of unknown concentration which were provided by the manufacturer. The negative cell control is the basal cytokine expression of the cells under normal growth conditions with no LPS. Each subset **(a-k)** is a graph of an individual cytokine as indicated on the x-axis. Y-axis is plotted on a logarithmic scale to allow for viewing a large range of values on a single plot, but the results have not been altered. Additional plots on a linear scale are displayed in S4 and S5 Figs.

To fully evaluate the potentially broad effects that the physical presentation method of LPS to cells, may have on cytokine expression, a multiplex assay kit that evaluated a larger panel of inflammatory cytokines (25 total) at increased sensitivity was used to determine the difference in patterns of cytokine expression between all three experimental conditions. The results for this assay are reported in fluorescence intensity. For this study, the pre-incubation step of LPS was eliminated in condition 2, to better mimic *in vivo* exposure conditions. As a parallel study, condition 3 was also evaluated, wherein LPS was presented in de-lipidated serum to determine the dynamic effects of the protective nature of intact serum carrier lipoproteins on pro-inflammatory cytokine expression. The difference in cytokine expression between conditions 1, 2, and 3, where LPS was added to the matrix (serum-free media, serum supplemented media, and media with de-lipidated serum) immediately prior to exposure to cells is shown in Fig 4. An alternative graph on a linear y-scale is presented in S4 Fig, and additional cytokines for these experiments can be reviewed in S5 Fig.

The induction of several pro-inflammatory cytokines is markedly reduced to near-baseline levels when LPS is presented in serum, even though the antigen was added to serum immediately prior to the experiment. This indicates rapid uptake of LPS into serum carrier proteins, and is in contrast to the results found by van Bergenhenegouwen *et*. *al*. [33] where extensive pre-incubation of LPS with serum lipoproteins was required to attenuate TLR4-induced cytokine expression. This can be explained by the varying susceptibility to endotoxin between mice and humans, [4, 57] in addition to the effect of stressful environmental conditions on the susceptibility of mice to septicemia, [58] which cannot be discounted when using commercial sera, as in condition 2. When comparing conditions 2 and 3, cytokine induction was consistently higher when LPS was presented to cells in de-lipidated serum (condition 3) than in serum (condition 2). This observation was universal for all LPS-related cytokines displayed in Fig 4a–4k, though some exceptions were noted for TLR4 independent cytokines (S5 Fig). The removal of serum lipids, and consequent inactivation of lipoprotein carriers such as HDL and LDL restores the pro-inflammatory cytokine response seen in condition 1, abrogating the protective effect of serum. [23, 27] These findings suggest that serum lipoprotein association is a significant factor in the protection of cells from the inflammatory effects of LPS. The amphiphilicity of PAMPs and their association with serum carrier proteins is a common theme in host-pathogen biology, begging the question regarding the implication of host carriers as protective forces for minimizing virulence.

**Fig 5:**
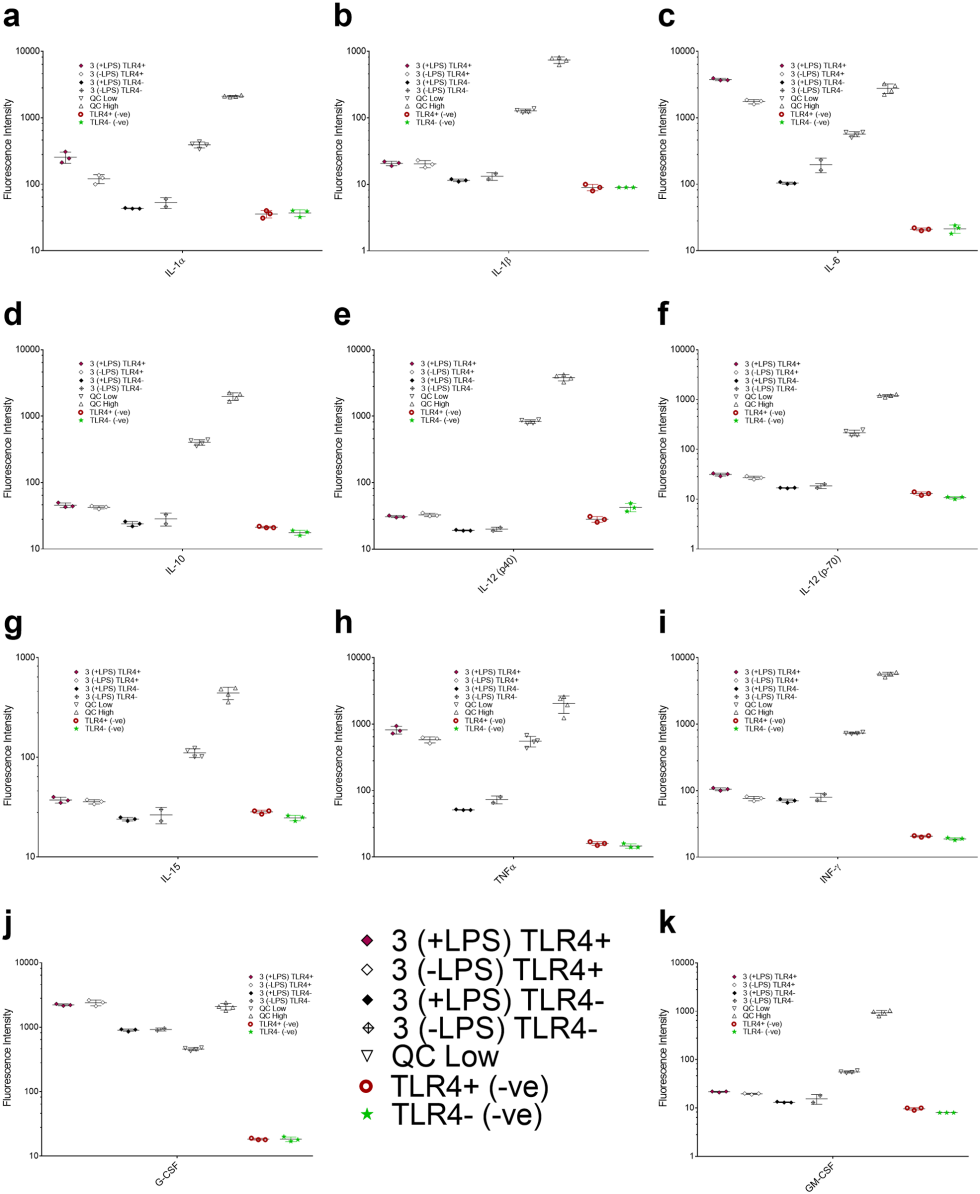
Comparison of cytokine response in condition 3 between TLR4(+) cells and TLR4(-) cells. Results of three independent replicates, plotted as fluorescence intensity for each individual cytokine, with error bars indicating the standard deviation of the mean for each condition. QC High and QC Low were high and low quality controls of unknown concentration, which were provided by the manufacturer. The negative cell controls indicate basal cytokine expression of the cells in normal growth conditions with no LPS. Each subset (a-k) is a graph of an individual cytokine as indicated on the x-axis. Y-axis is plotted on a logarithmic scale to allow for viewing a large range of values on a single plot.

De-lipidated serum can induce cellular stress, which in itself can contribute to inflammatory signaling. [59, 60] To discriminate between LPS-mediated, TLR4 dependent signaling and other stress-induced responses, we compared cytokine expression in TLR4(+) and TLR4(-) cell lines (Fig 5 and S6–S8 Figs) when LPS was presented in de-lipidated serum (condition 3). In this study, the TLR4(+) cells were SV40 transformed peritoneal macrophages, [61, 62] whereas the TLR4(-) cells were bone marrow derived macrophages from TLR4 knockout mice. [63] Different cell lines from varied progenitors can behave differently and may present varying biochemical characteristics and cytokine expression in basal and activated states. [64] However, in this study, we simply examine differential profiles under conditions 1, 2, and 3 in each of these cell lines to evaluate potential for TLR4-independent signaling, and stress induced response, which may impact outcomes.

An increase was noted in several TLR4-dependent cytokines when tested using de-lipidated serum, which can be attributed to a stress-induced response. [65] This induction was seen in both TLR4(+) and TLR4(-) cells, suggesting that it was not LPS-specific induction (Fig 5), but instead a response of the cell populations to deprivation of supportive serum components. We noted higher induction of IL-1α, IL-6, and GM CSF in the TLR4(+) cells with LPS, as compared to the TLR4(-) cells (Fig 5a, 5c and 5k), which helped to quantify the LPS-TLR4 dependent aspect of this response. The induction of pro-inflammatory cytokines suppressed in condition 2 was almost entirely restored in condition 3, and in many cases even exceeded the levels of expression seen in condition 1 (Fig 4b–4d, 4f and 4h–4k), further supporting a protective role for native lipoprotein carriers in serum in this response. In Fig 5, whereas the differential cytokine profiles are highlighted effectively, the numerical difference in LPS stimulation under all three conditions, may be difficult to infer from this representation. To this end, we have included all raw data in S1 Appendix, as well as an additional graph on a linear y-axis (S8 Fig).

**Fig 6:**
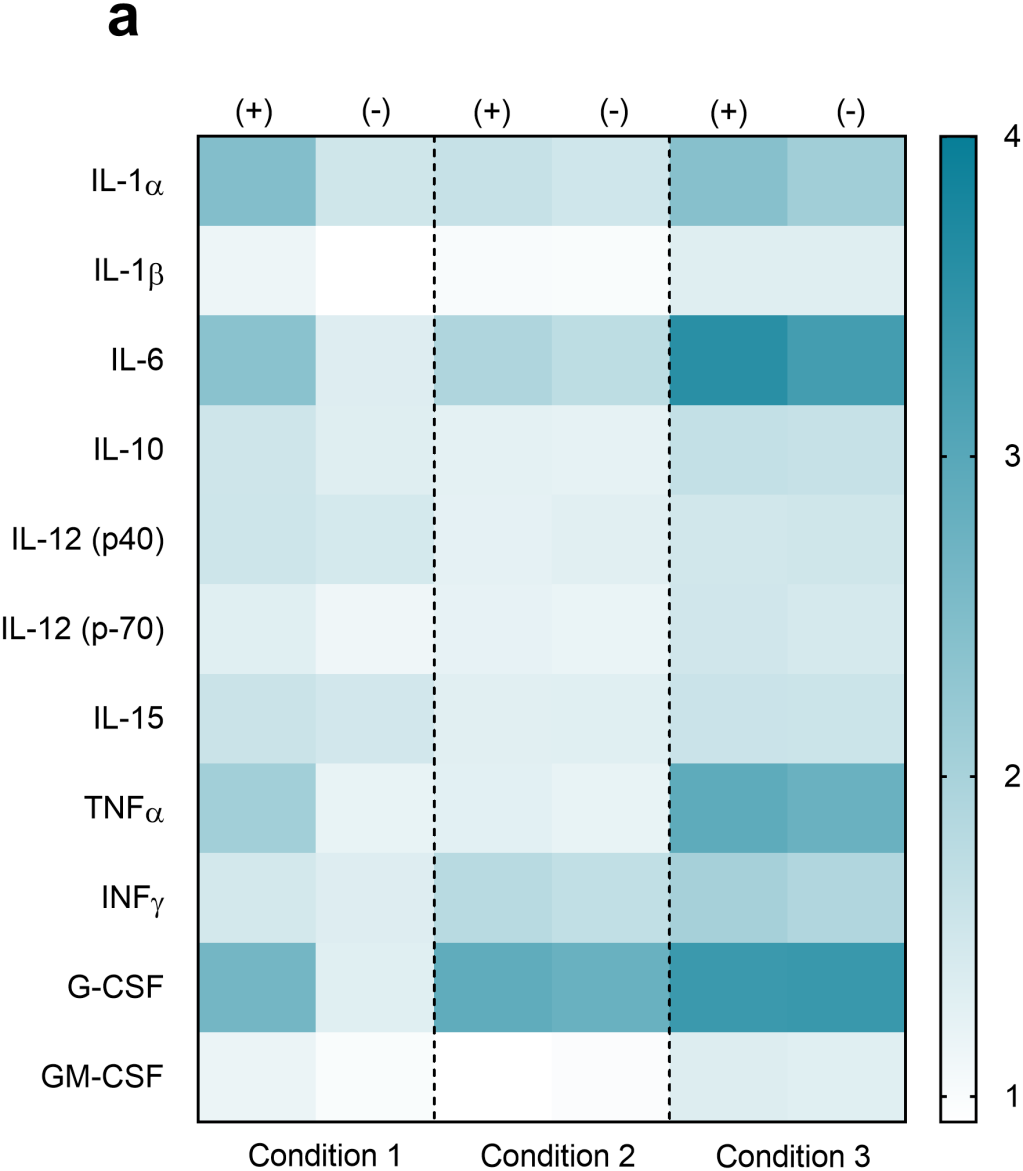
Heat map distribution of cytokines in each condition. Displays intensity profile of cytokine expression in TLR4(+) cells in conditions 1, 2, and 3, both with (+) and without (-) LPS stimulation. LPS condition is labeled on the upper axis, while serum condition is labeled on the lower axis. Scale bar indicates that 1 is the lowest (white color) and 4 is the highest (dark teal) intensity. Values were plotted as the mean value of the base 10 logarithm of median fluorescence intensity, n = 3.

Interestingly, a significant induction of the chemokines keratinocyte chemo-attractant (KC) was observed in TLR4(-) cells exposed to condition 1 (+LPS) and condition 3 in both the presence and absence of LPS (S6 and S7 Figs). Though we would like to note that in condition 2 (-LPS) and condition 3 (-LPS) a plating error during the fluorescence assay caused a repeat to be eliminated from each data set making it difficult to determine the true significance of these differences in these two specific cases. Regardless, since the TLR4(-) is only being used as an indicator of differential cytokine expression in the TLR4(+) cell line, the relative difference in expression can still be easily deduced, as in most cases presented in Fig 5, the levels of expression are near the baseline. KC belongs to a family of chemokines that cause leukocyte migration, and neutrophil recruitment as a result of inflammation, thus its presence in these conditions and control groups can be attributed to some degree of cellular stress. [40] It was also observed that G CSF was again found to be upregulated in the TLR4(-) cells (S6 Fig) as well as MCP-1 (a.k.a. CCL2) in condition 3 (S7 Fig) as compared to the negative cell control. MCP-1 has been demonstrated to serve as a chemo-attractant for memory T lymphocytes and NK cells during inflammation, and despite redundant cytokine signaling is solely responsible for mononuclear cell infiltration in murine models [66] Additionally of note were the increased levels of IFN-γ and IP-10 in this cell line in conditions 2 and 3, as compared to condition 1. As IP-10 is typically induced by IFN-γ, which appears to be present as similar levels in serum, both with and without LPS. However, these levels cannot be solely explained by contaminants in serum as a IFN-γ was increased in all conditions in the TLR4(+) cells (except condition 1 (-LPS)) using identical serum preparations, and thus the concurrent expression of IP-10 is also seen. In the case of Macrophage Inflammatory Proteins (MIP-1α, MIP-1β and MIP-2), we observed a pattern of high expression in the negative control cells for both MIP-1’s, yet low levels in MIP-2 (S5 Fig). However, when evaluating the difference between various conditions, it becomes quite clear that condition 2 has a demonstrably lower level of MIP-1’s indicating either the protective effect of serum, or possibly that native serum has co-factors or proteins that associate with MIP-1’s making them harder to detect in immunoassay type platforms. This becomes especially true when evaluating the negative cell control for these groups, as in both cases for MIP-1, it is markedly higher than condition 2. This trend was also similar for MIP-2, however the negative control is equivalent to that seen in condition 2, as well as condition 1 (-LPS) indicating that the expression witnessed in conditions 1 and 3 is markedly more significant. These observations could suggest presence of trace concentrations of LPS in cell media, which has been reported previously, [67] though the media used here was screened by the manufacturer for endotoxin content. Also, the response is not seen in condition 2 due to the protective effect of the serum carrier lipoproteins HDL, LDL, or possibly VLDL. We can also eliminate the possibility of LTA contamination, as the TLR4(-) cells are known to express 5-6x the amount of MIP-2 and TNFα in the presence of LTA, which is not exhibited here (S6 and S7 Figs). [63] Another possible explanation for this is due to the culturing method of the TLR4(-) cells, which requires the supplementation of sterile filtered LADMAC media to provide necessary growth factors for the cells. While the cells were rinsed thoroughly prior to testing, residual growth factors and cytokines in the TLR4(-) media could be present. The critical observation of these studies is that irrespective of the stress-induced cytokine and chemokine production observed in TLR4(-) cells, there is significant induction of LPS-specific cytokines in TLR4(+) cells in the absence of serum. This also demonstrates that the removal of serum lipoproteins largely eliminates the inflammatory suppression and protection observed in intact serum, as demonstrated in the heatmap analysis (Fig 6), where an intense response is noted for many of the TLR4-dependent cytokines for conditions 1 and 3 as compared to condition 2. The various patterns of differential cytokine expression observed with de-lipidation in TLR4(+) and TLR4(-) cells warrant further investigation, as they can shed critical information on alternative pathways and interaction of various biological processes *in vitro*.

The heat map (Fig 6) simply documents the changes discussed above in a collated format. Here, the scale ranges from 1–4, with 1 (white) being the lowest level of relative cytokine expression, and 4 (dark teal) representing the highest expression levels. The heat map indicates a significant increase in cytokine expression between conditions 1 and 3, as compared to condition 2. Specifically, condition 1 shows a dramatic induction in cytokine expression for IL-1α, IL-6, TNFα, and G CSF. Minimal induction of many cytokines is also seen condition 1, but this is also observed in the absence of LPS, and hence more associated with baseline signatures.

Colony stimulating factors, such as G CSF are linked to both survival and differentiation of macrophages. [65, 68]. In our experiments, upregulation of this chemokine is seen in conditions 1, 2 and 3, which may be associated with highly contradicting cues such as environmental stress, and cell growth and survival. Small quantities of LPS (0.1 ng/mL) have even been documented to aid in cell survival by causing production of CSF-1, though the effect is lost when concentrations are increased to microgram quantities. [68] Since G-CSF is often associated with stress-induced responses, upregulation could simply be a result of that stressor (de-lipidation), rather than specific to LPS in this case. Thus, while in condition 1, presence of LPS is mostly likely is the reason of upregulation of G CSF, this is clearly not the case in conditions 2 and 3. Under these conditions, G-CSF upregulation is seen both in the TLR4(+) and TLR4(-) cell lines (Fig 4 and S6 Fig), and in the presence and absence of LPS, which indicates that environmental stress and cell survival are the likely causes for this induction as shown in Fig 4j. This is yet an important indicator that the delivery method of LPS to cells affects the outcomes of cell signaling.

We also noted that condition 3 demonstrated a slight increase in cytokine expression in both the control and LPS stimulation groups, which can be attributed to LBP-mediated induction of TLR response by the antigen. Since LBP is a protein, it remains unaffected by the de-lipidation process, and thus remains in its native, active conformation and can deliver LPS to the receptors to induce innate immune signaling. Immunoblot analysis (S11 Fig) for LBP in dilutions of mouse serum yielded positive results, confirming availability of this protein carrier in said matrices.

When comparing control and LPS stimulation for condition 2 (Fig 6), the pattern of cytokine induction was found to be nearly identical, indicating attenuation in cytokine response in the presence of LPS, as compared to conditions 1 and 3, in most cases. In condition 2, the key point is that the degree of induction of IL-1α, IL-6, TNFα are all decreased when LPS is presented in serum. We also noted very slight upregulation of IFN-γ in condition 1 in the presence of LPS, as compared to the negative control. This effect was also seen in conditions 2 and 3, which can happen regardless of presence or absence of LPS—suggesting that a non-lipidic component of serum may be responsible for this induction, as opposed to LPS.

In the case of IL-6, a LPS dependent response was measured, as the induction of this cytokine in the presence of LPS was higher in all three conditions, but especially condition 3. IL-6 is implicated in LPS-dependent signaling, but is also known to be readily-induced during cell stress, environmental stress, and any type of trauma to the physiological system. The attenuating effects of serum lipoproteins on IL-6 expression have been previously noted [69], and have even been noted in de-lipidated bovine serum albumin. [65] In our study, however, we do not evidence the protective effects of albumins in condition 3, in the absence of serum lipoproteins, and observe an induction in IL-6 expression under this condition as well. Thus, it even seems likely, that de-lipidation of the serum is facilitating increased activity and transfer of LPS to TLR4 via LBP, which is plausible given that HDL and LDL facilitate clearance through the liver. However, it is possible, that this response under condition 2 and 3, while strong, is independent of the added LPS and could be attributed to non-lipidic components of serum as well, which is supported by the data seen in S6 Fig, where we see induction of IL-6 in TLR4(-) cells, which have been shown to be irresponsive to LPS.

Since IL-6 is a cytokine that is primarily implicated in stress-induced responses in cell systems, this response is not completely unexpected. [70, 71] The heat map representation of condition 3 shows increase in expression of the LPS-induced cytokines seen in condition 1, which further supports our hypothesis. IL-1α and IL-6 levels are higher with LPS than without, although the difference is not dramatically seen in the heat map presentation due to the data being presented as the base 10 logarithm of the fluorescence intensity values. It can be derived from the figures presented elsewhere in the manuscript (Fig 4 and S4 Fig).

In S10 Fig, panel a, in TLR4(+) cells, LPS induces IL-6 in all three conditions with comparable intensity. The induction of IL-6 in TLR4(+) cells, especially in condition 3 (de-lipidated serum) can be associated with the stress induced under these conditions. In the absence of LPS, there is no induction in TLR4 (+) cells. In TLR4(-) cells, the picture is different (S6 Fig). We see induction of IL-6 under all three conditions, but the intensity of induction is greater in condition 2, than in the other two conditions. Further, induction of IL-6 is also seen in LPS-free experimental conditions. Thus, enhanced induction could be physiologically relevant, or could be associated with other components in serum (such as lipoteichoic acid or other components), which can influence the response. The two cells lines are significantly different in the intensity of their responses. So, comparing the intensity of the response under a given condition between the two different cell lines is not suggested. In this case, the TLR4(-) cells were used as a benchmark of differential expression in cells with and without a LPS receptor, rather than to quantify differences in expression between the two systems. The other cytokines induced in condition 3 are seen irrespective of whether LPS is present or not. Thus, the pattern of cytokine expression demonstrated by this heat map supports our overarching hypothesis that cytokine induction is affected by the presentation of the amphiphilic LPS, and changes according to whether it is presented with or without serum carrier proteins, and whether those proteins are in their native conformations such as with serum versus de-lipidated serum.

K-means clustering analysis was performed to determine distinct expression patterns in response to the presence of LPS (S10 Fig). The cytokines were clustered based on: (i) LPS exposure condition, and (ii) similar median log fluorescence intensity (log MFI). The method was used to divide the cytokines into four distinct groups based on their proximity in log MFI (S10 Fig). This plot is divided by condition, and represents both the control and LPS stimulation groups which helps identify trends between the two. Evaluation of condition 1 indicates a substantial increase in cytokines in clusters 1–3, confirming expression of cytokines commonly associated with TLR4 dependent signaling. Cluster analysis of condition 2 shows limited change and induction of cytokine expression, which supports our previous conclusions regarding the protective effect of serum carrier proteins (both lipoproteins, and proteins like LBP). Lastly, clusters 1–3 are considerably higher for condition 3 in the control group, compared to conditions 1 and 2, which may be indicative of stress-induced effect on the TLR4(+) cell line (Fig 4 and S5 Fig). Minimal induction of cytokines was observed when the control and LPS stimulation groups for condition 3 were compared.

Taken all together, our study highlights the importance of amphiphile configuration on outcome of cellular studies, and by extension physiological relevance. Association with carrier molecules affects PAMP interactions with host molecules and activates pattern recognition receptors, which then influences cytokine signaling.

## Materials and methods

### Cell lines

IC-21 (TLR4 (+), ATCC^®^ TIB-186^™^ *Mus musculus*) cells are known to display normal macrophage behavior, compared to RAW 264.7 cells, which are often used in similar studies, [40] but do not display normal phenotypic behavior. IC-21 cells were grown in Dulbecco’s modification of Eagle’s medium (DMEM, Corning Cellgro^®^) supplemented with 10% fetal bovine serum (FBS, Sigma). No antibiotics or anti-mycotics were used. A comparative TLR4 knockout murine macrophage cell line [23ScCr (ATCC^®^ CRL-2751^™^)] was specifically selected as it is unresponsive to LPS stimulation. [72] Media for 23ScCr cells were made with DMEM + 10% FBS + 20% LADMAC media. Media supplement was produced from LADMAC murine macrophages (ATCC^®^ CRL-2420^™^) grown in Eagle’s minimum essential medium (Corning Cellgro^®^) supplemented with 10% FBS, and centrifuged every 4 days to harvest supernatant which contains essential growth factors for 23ScCr cells. To minimize potential for mycoplasma contamination, harvested media was filtered using Autofil 0.1 μm vacuum flasks (USA scientific). Both IC-21 and 23ScCr cell lines were cultured to ~90% confluence and split 1:4 as needed. During pass seven, cells were harvested and plated at a density of 1.0x106 cells per well in 12-well plates (Costar^®^), and incubated for 24 h at 37°C, 5% CO_2_. Media was refreshed, and the cells were incubated an additional 24 h. On the day of the experiment, cells were rinsed two times with serum free media prior to LPS exposure. All assays were performed in triplicate using independent wells.

### Measurement of critical nicelle concentration (CMC)

The CMC of *E*. *coli* O111:B4 LPS was determined using the Detergent Critical Micelle Concentration (CMC) Assay Kit (Profoldin, CMC1000). LPS from *E*. *coli* O111:B4 (Sigma Aldrich L2630) was resuspended to 5 mg/mL in sterile Nanopure water, followed by further dilution of LPS to 80 μg/mL in phosphate buffered saline (PBS, Sigma Aldrich). Low-retention (siliconized) tubes and pipet tips were used to avoid LPS sticking to the plastic surfaces. The CMC dye from the kit (1000x) was diluted to 1x in PBS, and 50 μL was combined with 50 μL of LPS (diluting 1:1), and incubated at room temperature for 30 min. Control wells did not contain LPS. Fluorescence measurements were taken using a SpectraMax Gemini EM plate reader (Molecular Devices, λ_ex_ = 355 nm, λ_em_ = 460 nm). The fluorescence ratio of LPS/no LPS was calculated and averaged for each concentration (n = 6) and represented as the mean with standard deviations between the replicates. The CMC was indicated by the inflection point at which the fluorescence ratio increased by a statistically significant amount (*p* < 0.05, Student’s *t*-test, two-tailed, equal variance) S1 Appendix.

### Evaluation of HDL and LDL in serum

The levels of HDL and LDL were measured in 100% human serum (Fisher CELLect^®^ BP2657100) and 100% de-lipidated human serum (Valley Biomedical HS2001). Controls consisted of PBS(-ve) and PBS(+ve) with 50 μg/mL *E*. *coli* O111:B4. 96-well microtiter plates (Corning, 9017) were coated with 100 μL of serum, PBS or 50 μg/mL LPS and incubated overnight at 4 °C. Wells were blocked with 200 μL PBS/0.05% Tween-20/0.5% bovine serum albumin for 1 h at room temperature (RT), then washed three times with PBS/0.5% Tween-20. Primary antibodies, α-apoAI (α-HDL, abcam 27630) or α-apoB (α-LDL, abcam ab20898), were diluted to 4 μg/mL in PBS, added to wells, and incubated for 1 h at RT. Wells were washed three times with PBS/0.5% Tween-20, and then a 1:1000 HRP-conjugated rabbit α-goat IgG antibody diluted in 1x PBS (SouthernBiotech 6160–05), was applied to the wells, covered and incubated for 45 min at RT. Wells were washed four times with PBS/0.5% Tween-20, and 100 μL of 1-Step^™^ Ultra TMB-ELISA Substrate Solution (Thermo Scientific 34028) was added to each well and incubated at 37 °C to aid in color development. The development was stopped by adding 200 μL of H_2_SO_4_ (2 M) and the absorbance was measured at 450 nm using a VersaMax plate reader (Molecular Devices). The average absorbance at 450 nm for each condition was measured (n = 6) and reported as the mean with standard deviations. A_450_ test values were compared to the PBS control (* *p* < 0.001, Student’s *t*-test, two-tailed, equal variance), S1 Appendix.

### Measurement of background LPS in serum

LPS is often a contaminant in serum, and presence of the antigen in mouse serum may affect the outcome of experiments. To determine this, mouse serum (Sigma Aldrich), human serum, and de-lipidated human serum were assessed using an enzyme-linked immunoassay. To measure the background LPS levels in serum, 96-well microtiter plates (Corning, 9017) were coated with 100 μL of mouse serum, human serum, or de-lipidated human serum. A positive control of 50 μg/mL LPS, and a negative control of PBS were used in place of serum and the microtiter plate was incubated for 45 min at RT, then rinsed 3x with PBS. Plates were blocked with 200 μL PBS/0.05% Tween-20/0.5% bovine serum albumin for 30 min at RT, then washed three times with PBS/0.5% Tween-20 before adding 100 μL of 40 μg/mL of α-LPS polyclonal antibody (Thermo Fisher PA1-7244) prepared in PBS/0.05% Tween-20/0.5% bovine serum albumin, which was incubated for 15 min at RT. Plates were washed three times with PBS/0.5% Tween-20 and 100 μL of HRP-conjugated goat α-rabbit IgG antibody (1:1000 in PBS/0.05% Tween-20/0.5% bovine serum albumin (SouthernBiotech 4055–05)) was added to each well and incubated for 15 min at RT. Wells were washed four times (PBS/0.5% Tween-20) and 100 μL of 1-Step^™^ Ultra TMB-ELISA Substrate Solution (Thermo Scientific 34028) was added to each well. The reaction was stopped by adding 200 μL of H_2_SO_4_ (2 M) and absorbance at 450 nm was recorded using a VersaMax plate reader (Molecular Devices). The average A_450_ for each condition was determined (n = 6), and standard deviations of the mean were calculated. A_450_ test values were compared to the PBS control (* *p* < 0.001, Student’s *t*-test, two-tailed, equal variance), S1 Appendix.

### Measuring the LPS induced response in IC-21 cells

A 12-plex mouse inflammatory cytokine multi-analyte ELISArray^™^ kit (Qiagen^®^) paired with LPS O111:B4 (phenol extract, Sigma Aldrich) was used to determine how many hours of LPS exposure was required to produce a measurable cytokine response. Limit of detection for the ELISAarray 12-plex kit is stated by the manufacturer as 10 pg/mL for each given cytokine. For further in-depth examination we chose ULTRA PURE LPS O111:B4 (List Labs) to avoid contaminates often found in other LPS preparations. [73] The difference in response between conditions was measured with a 25-plex Milliplex^®^ MAP mouse cytokine/chemokine Magnetic Kit (EMD Millipore, detection range 3.2–10,000 pg/mL).

Thus, we have chosen to use both an absorbance and a fluorescence-based assay for the measurement of cytokine and chemokine expression in this manuscript. The absorbance-based measurements were performed using a Qiagen Multi-Analyte ELISArray Kits that can profile 12 cytokines in complex samples such as cell supernatant (media), serum, or plasma samples using one simple protocol and one standardized development time. The kit utilizes commercially available capture and detection antibodies to achieve the highest possible sensitivity and linearity for each cytokine or chemokine assay. The kit uses a standard sandwich ELISA protocol and is compatible with a standard ELISA plate reader, and is effective only over a limited concentration range for each cytokine, beyond which the signal saturates making quantification of outcomes difficult, without multiple dilutions, making continual testing cost-prohibitive. However, the assay is limited to only 12 cytokines, and the sensitivity of detection is an order of magnitude less than the fluorescence measurement kits (Limit of detection for the ELISA array 12-plex kit is stated by the manufacturer as 10 pg/mL for each given cytokine). Therefore, a comprehensive measurement of the difference in response between conditions was measured with a 25-plex Milliplex^®^ MAP mouse cytokine/chemokine Magnetic Kit (EMD Millipore, detection range 3.2–10,000 pg/mL). This method simultaneously analyzes multiple (n = 25) cytokine and chemokine biomarkers with Bead-Based Multiplex Assays using the Luminex technology. In addition to greater coverage and enhanced sensitivity, this method is known to provide more reliable and reproducible measurements in complex samples.

For all cytokine studies, LPS stocks (5 mg/mL) were sonicated in a bath sonicator (Branson 2510) for 15 min, then diluted to 100 ng/mL in serum free media or (buffer), resonicated, and applied to wells. In all experiments involving LPS, the antigen was prepared and stored in glass or silanized tubes, which were also used for the preparation of the working dilutions. The dilutions were transferred to plastic tubes/well only immediately before the experiment to facilitate mixing larger volumes of media. For condition 2, sonicated LPS was spiked into mouse serum (Sigma Aldrich), vortexed intermittently for 2 min, and incubated overnight at 4°C to allow for association of LPS with lipoproteins, or alternately applied immediately to cells without overnight incubation. All cells were rinsed two times with serum free media before application of condition 1, 2, or 3 media. De-lipidated serum for condition 3 was prepared as described by the manufacturer (ImmunoReagents, Inc.) in endotoxin-free water (cell-culture grade, ThermoFisher). Negative control cells received DMEM + 50% mouse serum, but no LPS.

Positive and negative controls for all cytokines presented were provided by the manufacturer to ensure kit performance. Negative controls in the case of the Qiagen multiplex ELISArray consisted of antibody attached to the plate surface (provided pre-attached to the plate by manufacturer) and then incubated with sterile cell culture media, while other treatments received cell culture media that had been incubated with cells. Subsequently, plates were washed and incubated with the detection antibody prior to development. Positive kit controls were incubated with a prescribed dilution of each respective antigen (provided by manufacturer) in sterile cell culture media. Additional negative controls were added to evaluate any potential cytokine response in the cells in the absence of LPS, or with high concentrations of serum.

Except for the time course assays, cells were incubated (37°C, 5% CO_2_) for 8 h, after which media supernatant was collected and assayed for cytokine levels using either the 12-plex or 25-plex mouse cytokine/chemokine kits. Results for the 12-plex kit were measured on a SpectraMax M5 (Molecular Devices) and plotted as the mean absorbance values with standard deviations of the replicates. 25-plex cytokine results were obtained using a MAGPIX^®^ (Luminex), and processed as described below.

### Immunoblotting of serum for LBP

To assess the presence of LBP in dilutions of murine serum, whole mouse serum was diluted to 50% and 25% using PBS. 2 μL of each dilution of serum (100%, 50%, 25%), as well as 5% BSA, and PBS were blotted onto nitrocellulose membrane and allowed to dry. The membrane was then blocked with 2% BSA for 1 h, rinsed 3x with 0.1% Tween-20/PBS (5 min each) and then 3x with PBS (5 min each). The filter paper was then incubated with the primary antibody (1:2000 dilution in PBS, rat monoclonal α-LBP, (BEI Resources, Clone U54.R.mLBP.2)) for 1 h at RT, followed by rinsing, and incubation with 1:4000 Goat α-rat alkaline phosphatase (AbCam, ab6846) for 1 h, and development with 1-Step NBT-BCIP substrate solution (Thermo Fisher) for 20 min at RT.

### Data processing and statistical analysis

All statistical analyses, processing, and graphing of data sets was performed using GraphPad Prism 7, except for analysis of the k-means clustering which was performed using Matlab (R2017a). A K means clustering algorithm was used to cluster the cytokines into k clusters, by utilizing the square Euclidean distances. 12-plex cytokine assays were plotted as the mean of absorbance replicates (n = 3) with standard deviations. Analysis of 25-plex cytokine assays was performed using multiple methods. To demonstrate the variance of individual replicates the data was plotted as a cluster plot for each cytokine with standard deviations of the mean. Additionally, an asymmetric 5 parameter logistic function was applied to the mean fluorescence intensity values for the standard curve. The governing equations were then used to interpolate the fluorescence values of the unknowns to convert to Log[pg/mL]. As some of the MFI values for measured cytokines fell outside of the standard curve, they could not be properly interpolated into Log[pg/mL] and thus are displayed as blank spaces in S1 Appendix. These blank spaces inhibited statistical analysis and comparison between the different conditions, however, we present the values here for referencing, and inferring concentrations of cytokines. Additional graphs (S4 and S8 Figs) displayed in fluorescence intensity are graphed on a linear y-axis scale to enable careful evaluation of the variance between replicates. Results from 2-way analysis of variance (ANOVA) tests were performed to compare the mean value of a cytokine within each condition with LPS to that in the same condition without LPS, and results are displayed in S1 Appendix. Significance between cytokine levels was determine using α = 0.05 and Sidak’s multiple comparisons test. All raw data sets for cytokine studies, CMC experiments, HDL/LDL in serum examination, LPS contaminants in serum, ANOVA results, Grubb’s Outlier Tests, and regression analyses are provided for review in sheets found in S1 Appendix.

## Supporting information

S1 Fig Characterization of LPS. **(a)** Critical micelle concentration of LPS in 1x PBS. Data are reported as the mean fluorescence ratio between sample and control cases with error bars representing the standard deviation of the mean. The black bar indicates the range for LPS O111:B4 CMC in 1x PBS. **(b)** Characterization of HDL and LDL and **(c)** LPS in mouse serum, and **(d)** LPS in human serum and de-lipidated human serum (n = 6) for all three (b-d) experiments. Data are reported as the mean absorbance with error bars indicating the standard deviations.
(TIF)

S2 Fig Cytokine profile over time of 12 cytokines. Condition 1 system plotted as a bar plot with positive and negative kit controls to allow for side-by-side evaluation of results. Parameters of kit controls are described in the main text.
(TIF)

S3 Fig Cluster plot of cytokines of murine macrophages after 8 hours in systems under conditions 1 and 2 with and without LPS. Each subset **(a-l)** is a graph of an individual cytokine as indicated on the x-axis. Each replicate value is shown and plotted with the mean plotted as a line through the points. Positive and negative kit controls for each cytokine are plotted with the negative control simply being a well functionalized with capture antibody and then incubated with buffer versus the positive control where a manufacturer prescribed dilution of cytokine was added to each well. The (–LPS) conditions serve as additional negative controls to monitor the baseline cytokine expression of the cells with and without serum.
(TIF)

S4 Fig Data from Fig 4 plotted on a linear scale of fluorescence intensity. Each subset (a-k) is a graph of an individual cytokine as indicated on the x-axis. Cluster plot of results of three independent replicates, plotted as fluorescence intensity for each individual cytokine, with error bars indicating the standard deviation of the mean for each condition. QC High and QC Low were high and low quality controls of unknown concentration which were provided by the manufacturer. The negative cell control (-ve) is the basal cytokine expression of the cells in normal growth conditions with no LPS.
(TIF)

S5 Fig Additional cytokines and chemokines measured for conditions 1, 2, and 3, shown with and without LPS. Each subset **(a-k)** is a graph of an individual cytokine as indicated on the x-axis. Cluster plot of results of three independent replicates, plotted as fluorescence intensity for each individual cytokine, with error bars indicating the standard deviation of the mean for each condition. QC High and QC Low were high and low quality controls of unknown concentration which were provided by the manufacturer. The negative cell control is the basal cytokine expression of the cells in normal growth conditions with no LPS. Y-axis is plotted on a logarithmic scale to allow for viewing a large range of values on a single plot.
(TIF)

S6 Fig Cytokine response of TLR4(-) cells in the presence and absence of LPS stimulation using all three experimental conditions. Cluster plot of results of three independent replicates, plotted as fluorescence intensity for each individual cytokine, with error bars indicating the standard deviation of the mean for each condition. QC High and QC Low were high and low quality controls of unknown concentration, which were provided by the manufacturer. The negative cell control is the basal cytokine expression of the cells in normal growth conditions with no LPS. Y-axis is plotted on a logarithmic scale to allow for viewing a large range of values on a single plot.
(TIF)

S7 Fig Cluster plot for additional cytokine measurements for TLR4(-) cells under conditions 1, 2, and 3. Results of three independent replicates were plotted as fluorescence intensity for each individual cytokine, with error bars indicating the standard deviation of the mean for each condition. QC High and QC Low were high and low quality controls of unknown concentration, which were provided by the manufacturer. The negative cell control is the basal cytokine expression of the cells in normal growth conditions with no LPS. Y-axis is plotted on a logarithmic scale to allow for viewing a large range of values on a single plot.
(TIF)

S8 Fig Data from Fig 5 plotted on a linear scale of fluorescence intensity. Each subset (a-k) is a graph of an individual cytokine as indicated on the x-axis. Cluster plot of results of three independent replicates, plotted as fluorescence intensity for each individual cytokine, with error bars indicating the standard deviation of the mean for each condition. QC High and QC Low were high and low quality controls of unknown concentration which were provided by the manufacturer. The negative cell control is the basal cytokine expression of the cells in normal growth conditions with no LPS.
(TIF)

S9 Fig Standard curves for 25-plex assay kit. **(a-y)** Results of four independent replicates of manufacturer prepared standards were plotted as fluorescence intensity for each individual cytokine, with error bars indicating the standard deviation of the mean for each condition. In the case of G-CSF, GM-CSF, KC, RANTES, MIP-2, MIP-1B, and MIP1a, one of the values for 10,000 pg/mL presented as an outlier and was eliminated using Grubb’s Outlier test, with an alpha = 0.05. QC High and QC Low were high and low quality controls of unknown concentration, which were provided by the manufacturer. The negative cell controls were the basal cytokine expression of the cells in normal growth conditions with no LPS. Y-axis is plotted on a logarithmic scale to allow for viewing a large range of values on a single plot.
(TIF)

S10 Fig Distribution and clustering of all cytokines in each condition. **(a)** Heat-map intensity of cytokine expression in conditions 1, 2, and 3, both with (+) and without (-) LPS stimulation. LPS condition is labeled on the upper axis, while condition is labeled on the lower axis. Scale bar indicates that 1 is the lowest and 4 is the highest intensity. Values are plotted as the mean log MFI, n = 3. **(b)** K-means clustering of cytokine expression in all conditions, with and without LPS stimulation. Cluster 1 (Black) = MIP-1α, MIP-1β, and MIP-2. Cluster 2 (Blue) = IL-6, IP-10, and G CSF. Cluster 3 (Magenta) = IL-1α, MCP-1, TNFα, INFγ, and RANTES. Cluster 4 (Cyan) = GM CSF, IL-1β, IL-2, IL-4, IL-5, IL-7, IL-9, IL-10, IL-12 (p40), IL-12 (p70), IL-13, IL-15, IL-17, and KC.
(TIF)

S11 Fig Immunoblot of LBP in dilutions of murine serum. Dark spots indicate a positive result for all dilution of mouse serum and negative controls (5% BSA and 1x PBS) have no spots.
(TIF)

S1 Appendix Raw data and statistical analyses from cytokine experiments. Data is organized by sheet and described in the order that sheets are organized. The Time Point Study sheet contains absorbance data for TLR4(+) cells treated with condition 1 and measured at 4 hour intervals over a 24-hr period. The 24-hr Serum TLR4(+) sheet contains absorbance data for TLR4(+) cells that were treated with condition 2 serum that had been pre-incubated with LPS for a 24-hr period prior to exposing the cells. TLR4(+) data contains the cytokine measurements in median fluorescence intensity for the 25-plex studies of conditions 1–3 assessed in IC-21 murine macrophages. Similarly, sheet TLR4(-) contains 25-plex data for 23ScCr cells (wells in blue that are italicized and marked with an asterisk have been eliminated from the analysis due to misplating during the assay). Regression analysis for 25 plex contains the standard curve values for each cytokine as well as the 5 parameter logistic curves derived from the fluorescence values followed by the individual replicates interpolated into Log[pg/mL]. Data for this sheet ends at column BZ and row 91. Sheet “Condition 1 –Stats” contains the results of the ANOVAs as well as the Sidak’s multiple comparisons tests for all of the condition 1 cases analyzed. Data for this sheet ends at column AR and row 85. Sheets Condition 2 –Stats and Condition 3 –Stats are laid out in an identical manner as to the previous Condition 1 –Stats sheets and contain the data and analysis for the respective conditions. LPS CMC sheet contains the raw data for the calculation of the CMC of LPS in PBS. Data for this sheet ends at column P and row 52. HDL-LDL provides the data collected to determine the amounts of the lipoproteins in PBS, de-lipidated human serum, as well as human serum, and the positive control LPS. The next two sheets are labeled “LPS in mouse serum” and “LPS in human serum”. Both of these sheets provide the raw absorbance values and calculations for determining how much LPS is in PBS, the respective serums and the positive control LPS groups.
(XLS)
