# Supplementary figures and images for: Presentation matters: Impact of association of amphiphilic LPS with serum carrier proteins on innate immune signaling
Source: PLoS One. 2018 Jun 14;13(6):e0198531. doi: 10.1371/journal.pone.0198531 (PMC6002092; doi:10.1371/journal.pone.0198531)

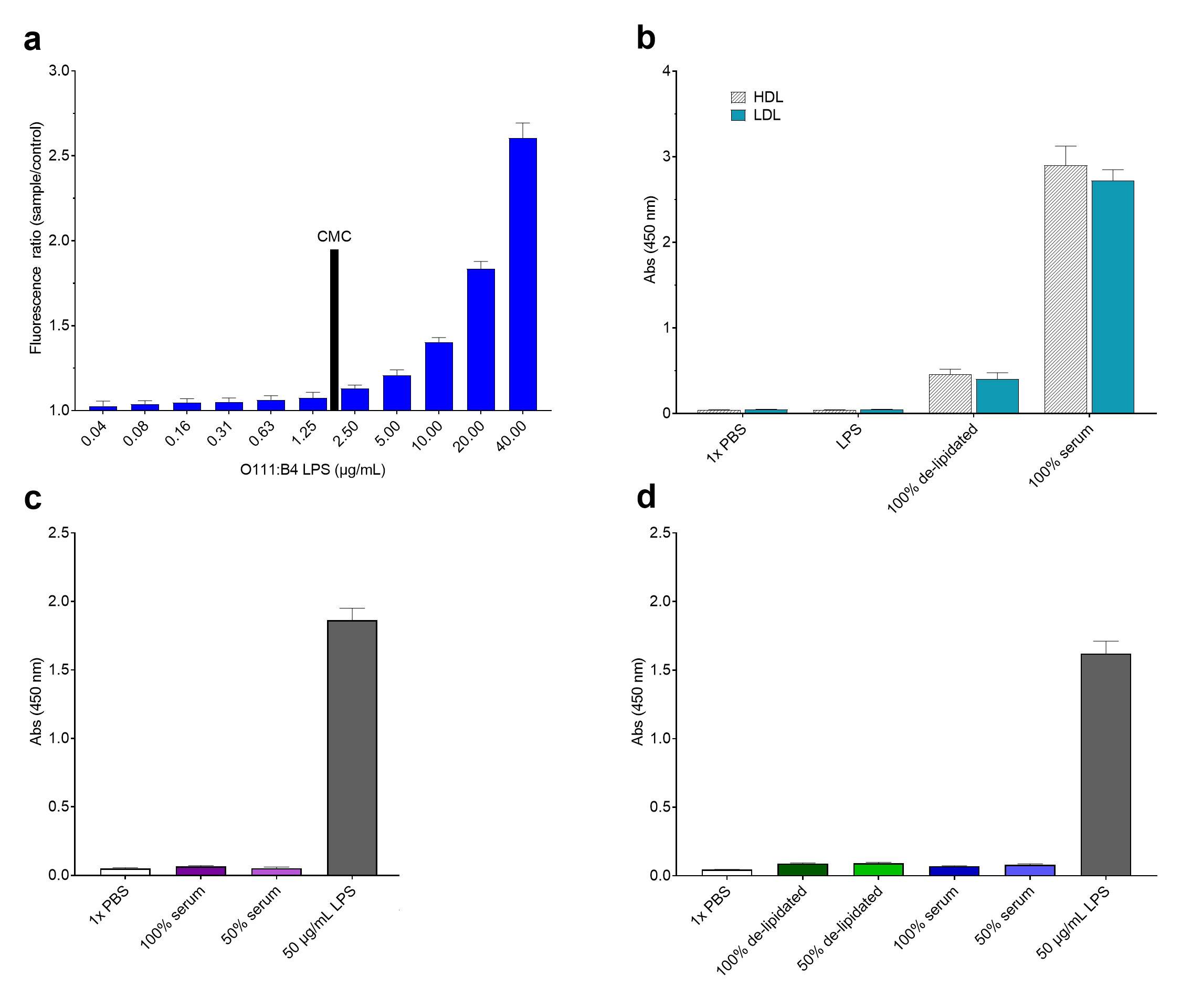

Supplement: S1 Fig — (a) Critical micelle concentration of LPS in 1x PBS. Data are reported as the mean fluorescence ratio between sample and control cases with error bars representing the standard deviation of the mean. The black bar indicates the range for LPS O111:B4 CMC in 1x PBS. (b) Characterization of HDL and LDL and (c) LPS in mouse serum, and (d) LPS in human serum and de-lipidated human serum (n = 6) for all three (b-d) experiments. Data are reported as the mean absorbance with error bars indicating the standard deviations. (TIF) [file pone.0198531.s001.tif]

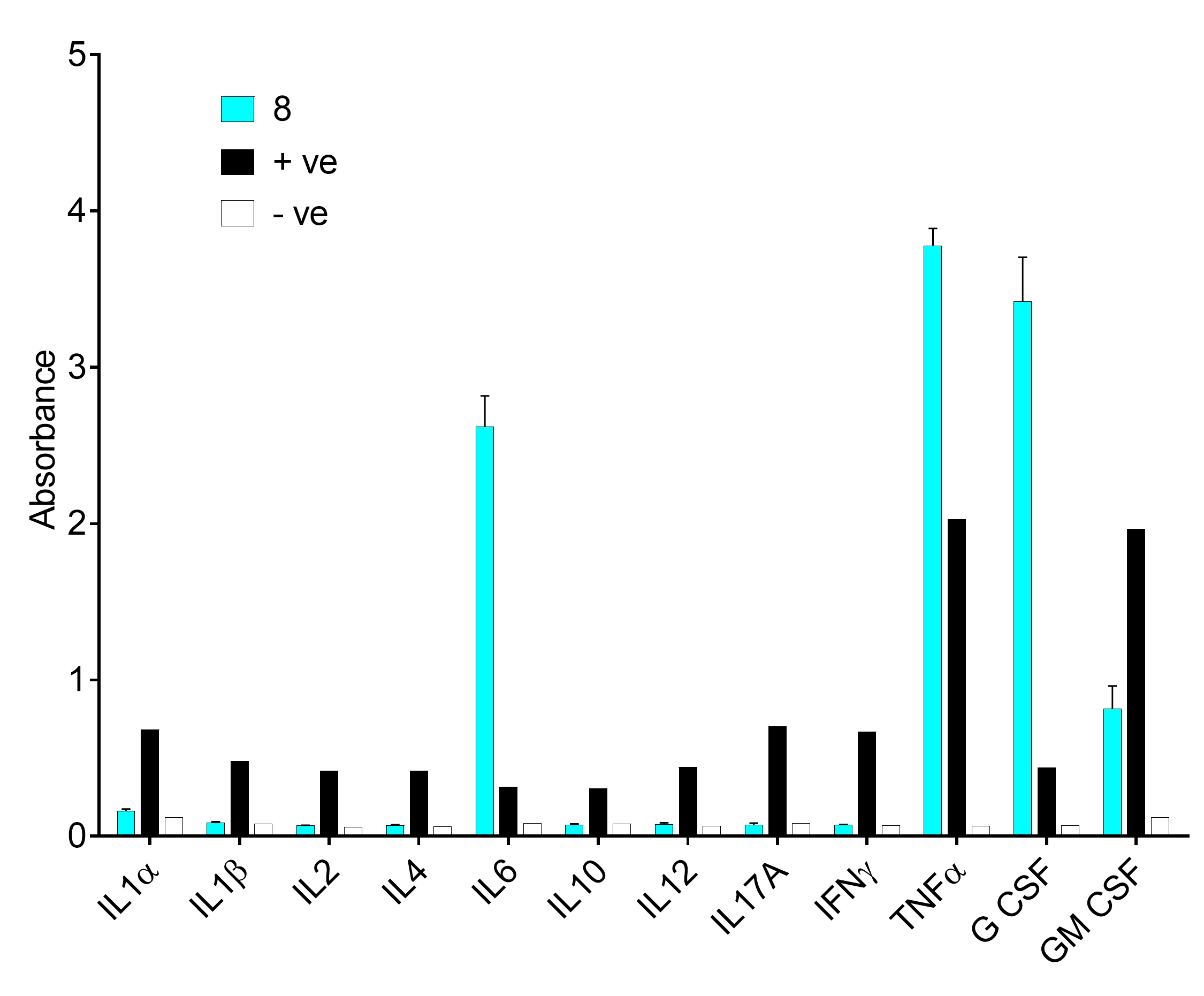

Supplement: S2 Fig — Condition 1 system plotted as a bar plot with positive and negative kit controls to allow for side-by-side evaluation of results. Parameters of kit controls are described in the main text. (TIF) [file pone.0198531.s002.tif]

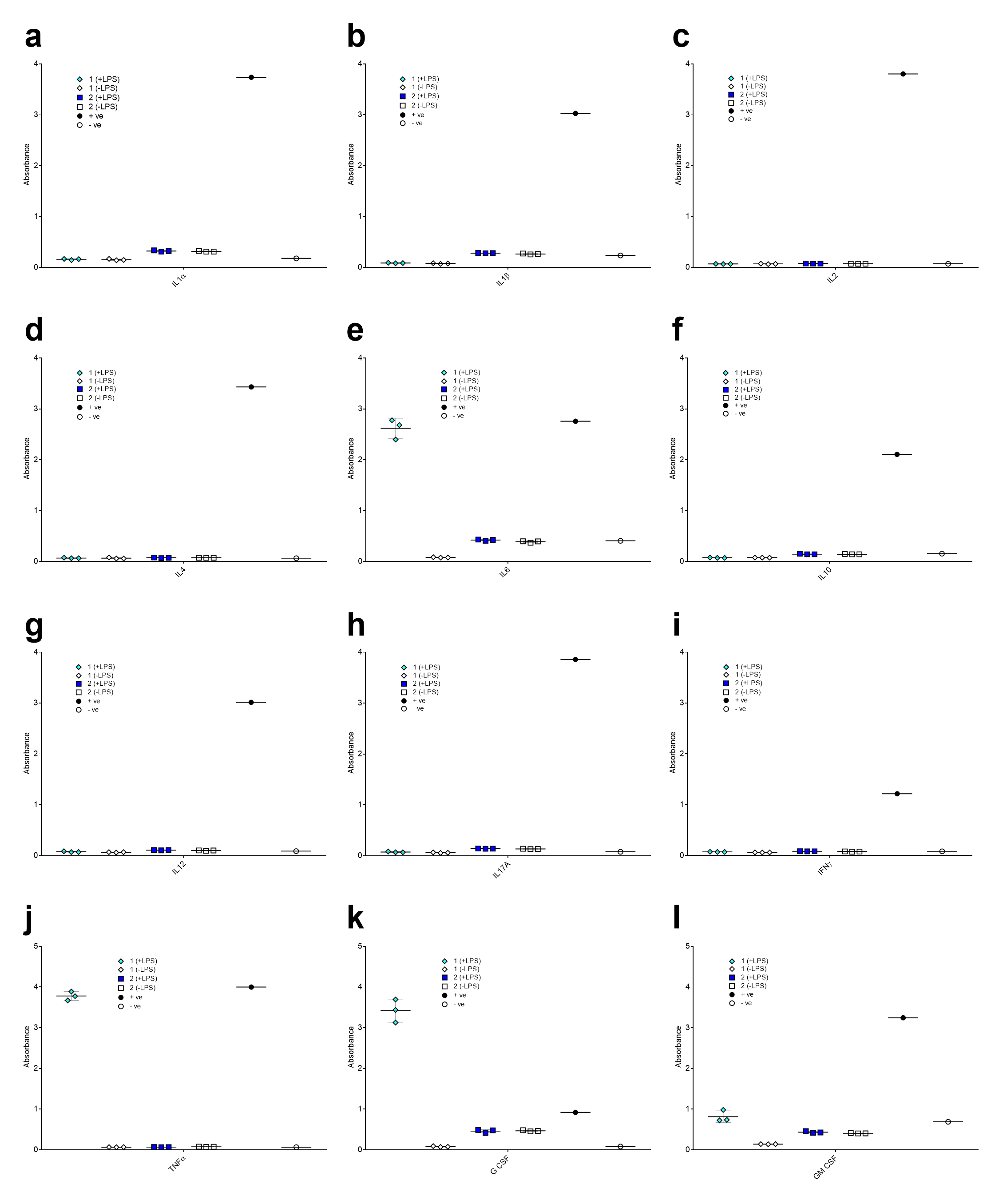

Supplement: S3 Fig — Each subset (a-l) is a graph of an individual cytokine as indicated on the x-axis. Each replicate value is shown and plotted with the mean plotted as a line through the points. Positive and negative kit controls for each cytokine are plotted with the negative control simply being a well functionalized with capture antibody and then incubated with buffer versus the positive control where a manufacturer prescribed dilution of cytokine was added to each well. The (–LPS) conditions serve as additional negative controls to monitor the baseline cytokine expression of the cells with and without serum. (TIF) [file pone.0198531.s003.tif]

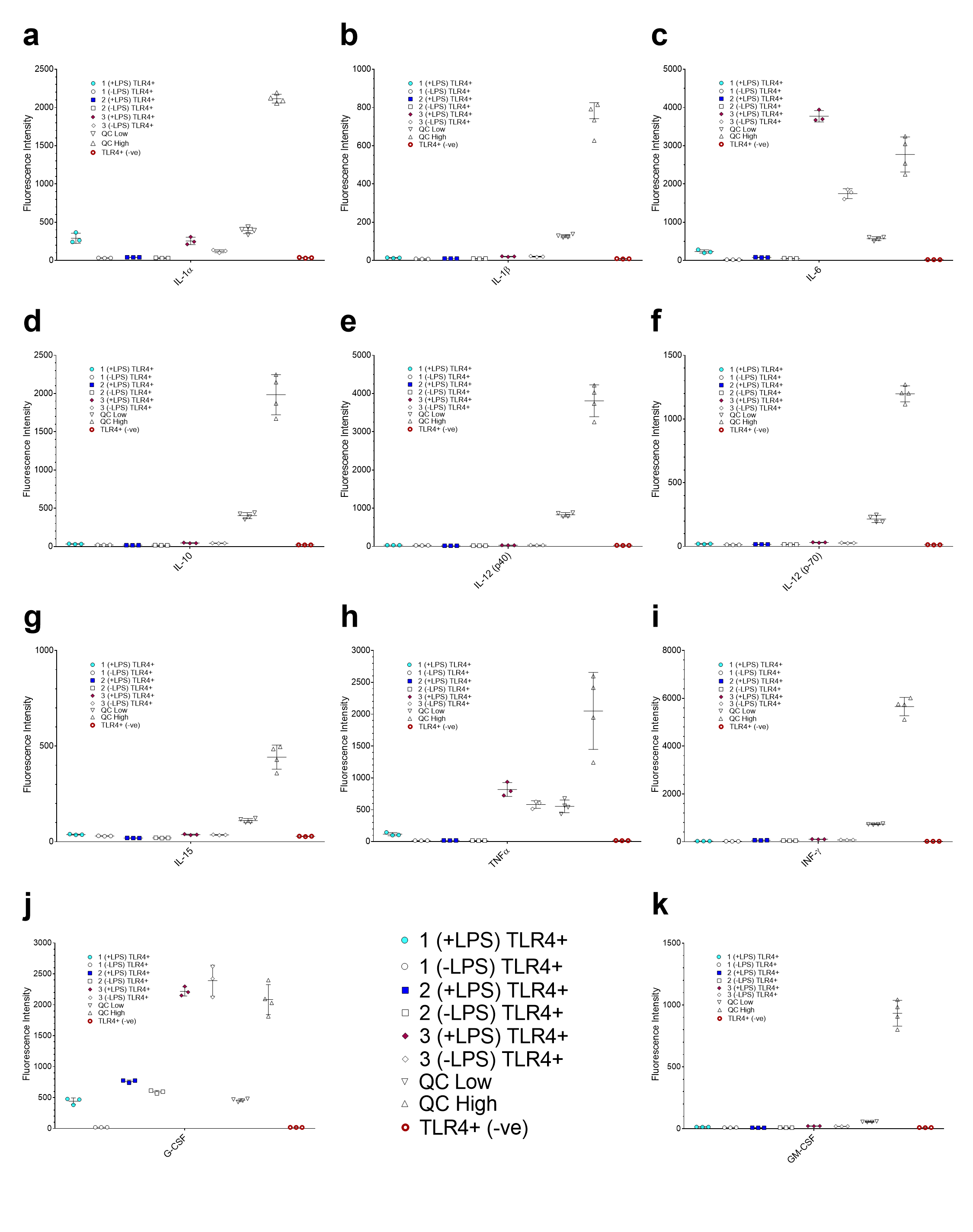

Supplement: S4 Fig — Each subset (a-k) is a graph of an individual cytokine as indicated on the x-axis. Cluster plot of results of three independent replicates, plotted as fluorescence intensity for each individual cytokine, with error bars indicating the standard deviation of the mean for each condition. QC High and QC Low were high and low quality controls of unknown concentration which were provided by the manufacturer. The negative cell control (-ve) is the basal cytokine expression of the cells in normal growth conditions with no LPS. (TIF) [file pone.0198531.s004.tif]

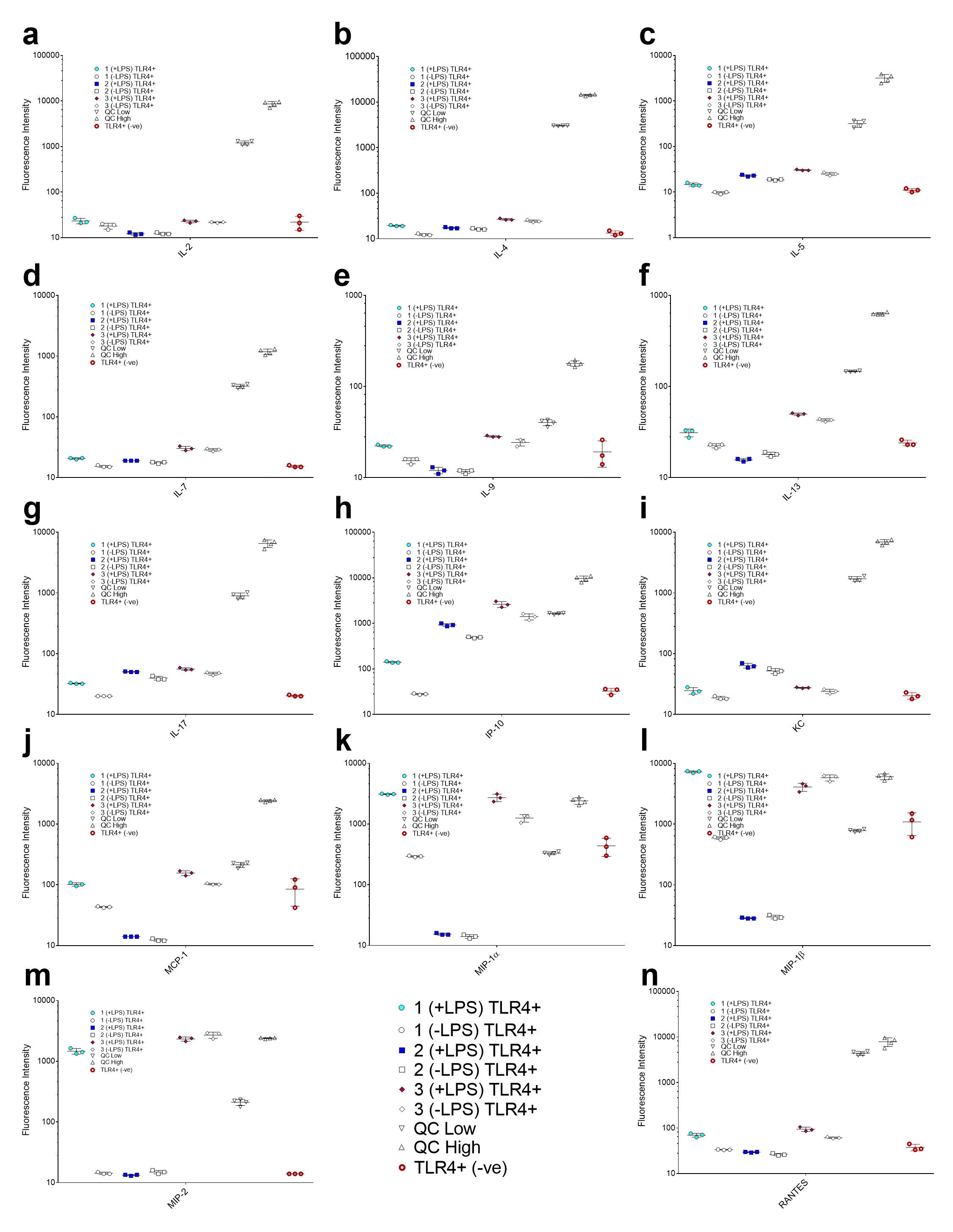

Supplement: S5 Fig — Each subset (a-k) is a graph of an individual cytokine as indicated on the x-axis. Cluster plot of results of three independent replicates, plotted as fluorescence intensity for each individual cytokine, with error bars indicating the standard deviation of the mean for each condition. QC High and QC Low were high and low quality controls of unknown concentration which were provided by the manufacturer. The negative cell control is the basal cytokine expression of the cells in normal growth conditions with no LPS. Y-axis is plotted on a logarithmic scale to allow for viewing a large range of values on a single plot. (TIF) [file pone.0198531.s005.tif]

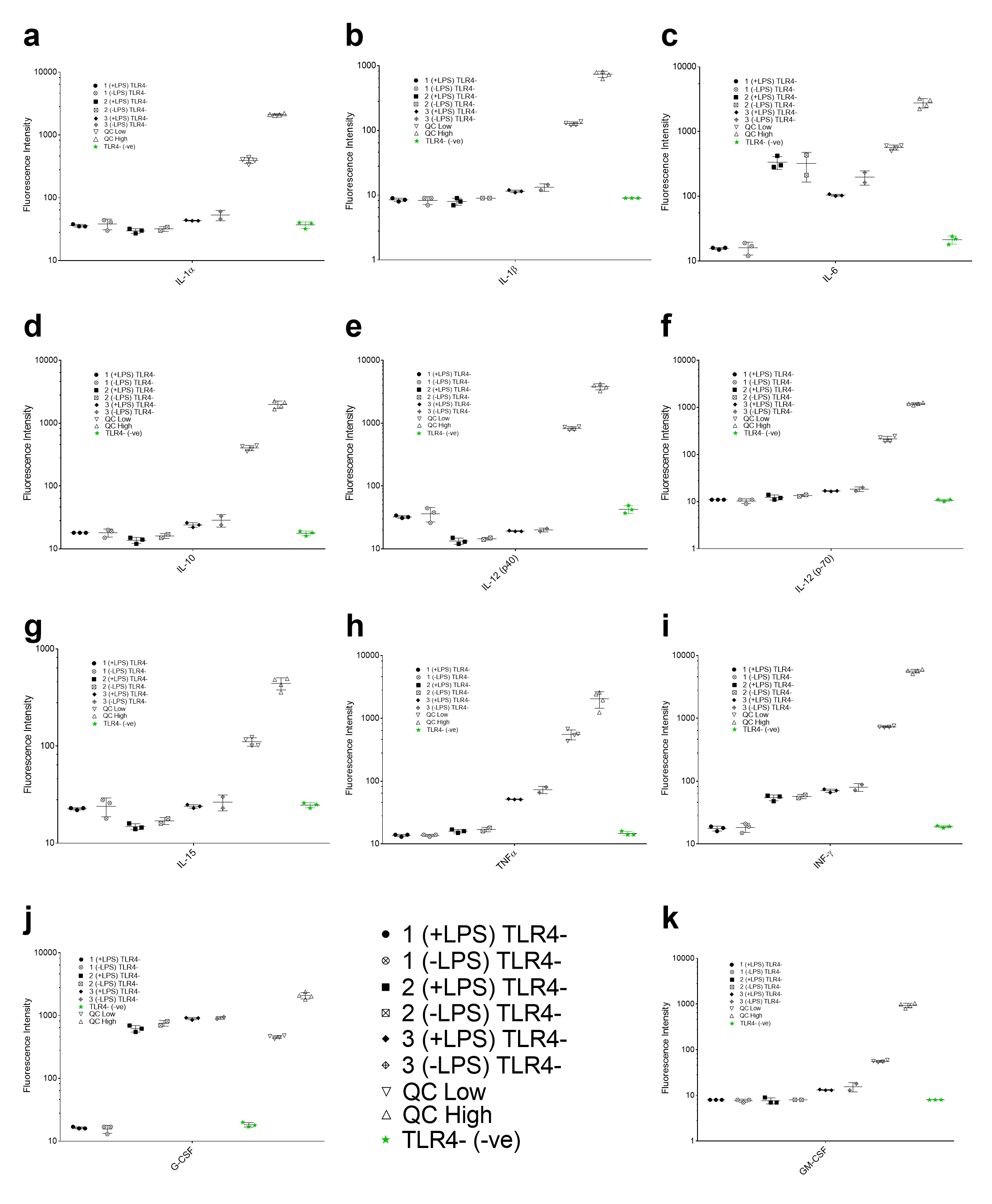

Supplement: S6 Fig — Cluster plot of results of three independent replicates, plotted as fluorescence intensity for each individual cytokine, with error bars indicating the standard deviation of the mean for each condition. QC High and QC Low were high and low quality controls of unknown concentration, which were provided by the manufacturer. The negative cell control is the basal cytokine expression of the cells in normal growth conditions with no LPS. Y-axis is plotted on a logarithmic scale to allow for viewing a large range of values on a single plot. (TIF) [file pone.0198531.s006.tif]

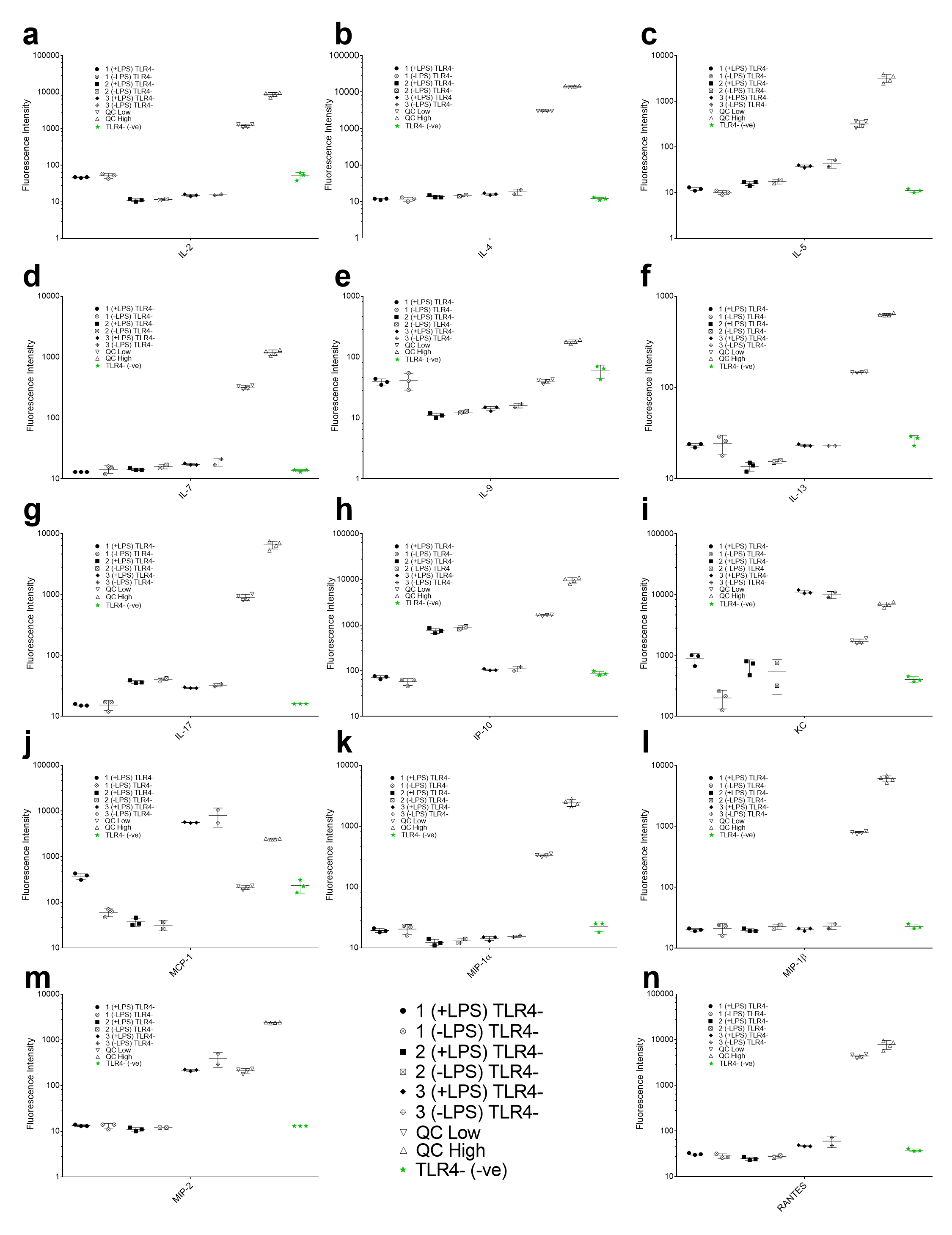

Supplement: S7 Fig — Results of three independent replicates were plotted as fluorescence intensity for each individual cytokine, with error bars indicating the standard deviation of the mean for each condition. QC High and QC Low were high and low quality controls of unknown concentration, which were provided by the manufacturer. The negative cell control is the basal cytokine expression of the cells in normal growth conditions with no LPS. Y-axis is plotted on a logarithmic scale to allow for viewing a large range of values on a single plot. (TIF) [file pone.0198531.s007.tif]

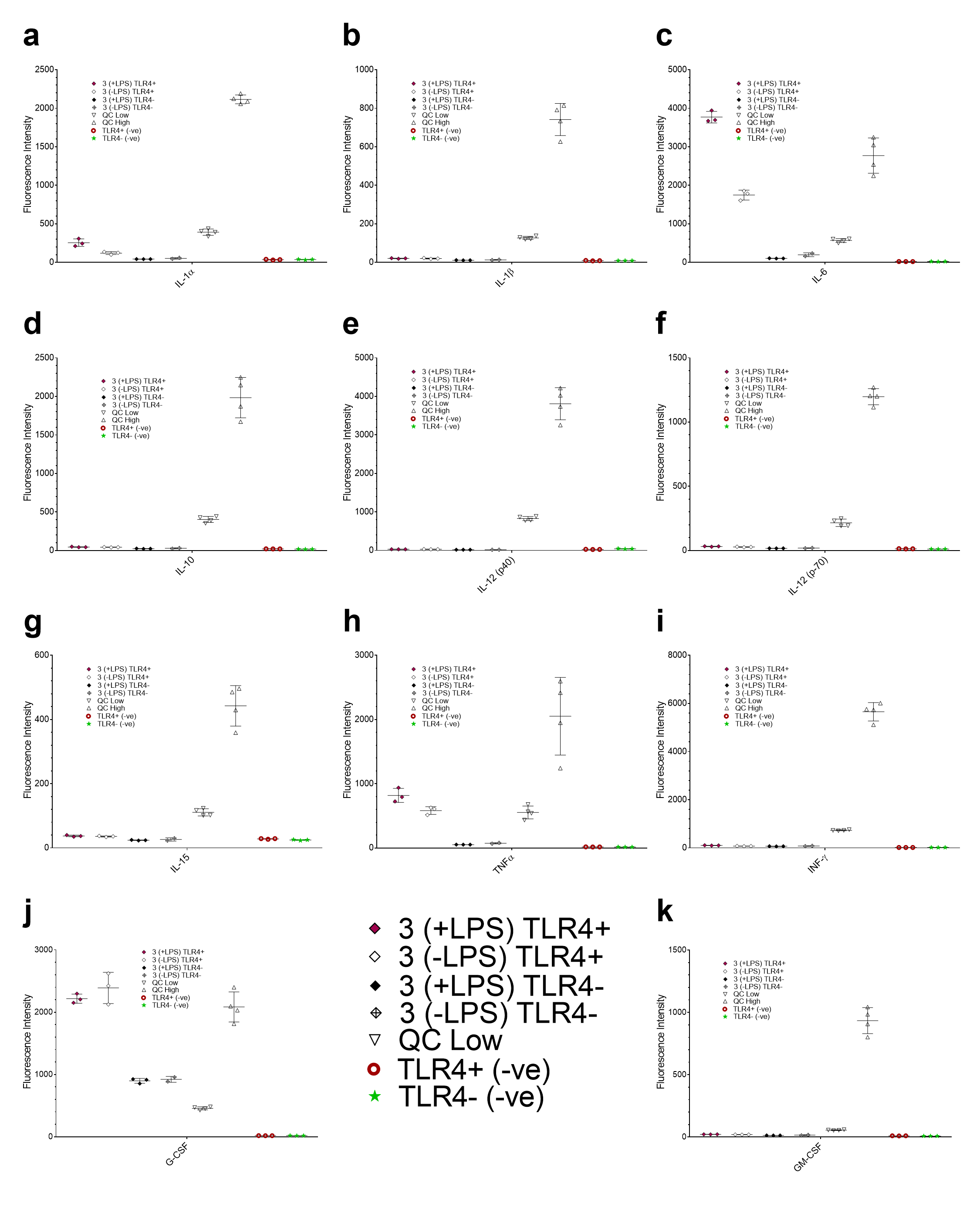

Supplement: S8 Fig — Each subset (a-k) is a graph of an individual cytokine as indicated on the x-axis. Cluster plot of results of three independent replicates, plotted as fluorescence intensity for each individual cytokine, with error bars indicating the standard deviation of the mean for each condition. QC High and QC Low were high and low quality controls of unknown concentration which were provided by the manufacturer. The negative cell control is the basal cytokine expression of the cells in normal growth conditions with no LPS. (TIF) [file pone.0198531.s008.tif]

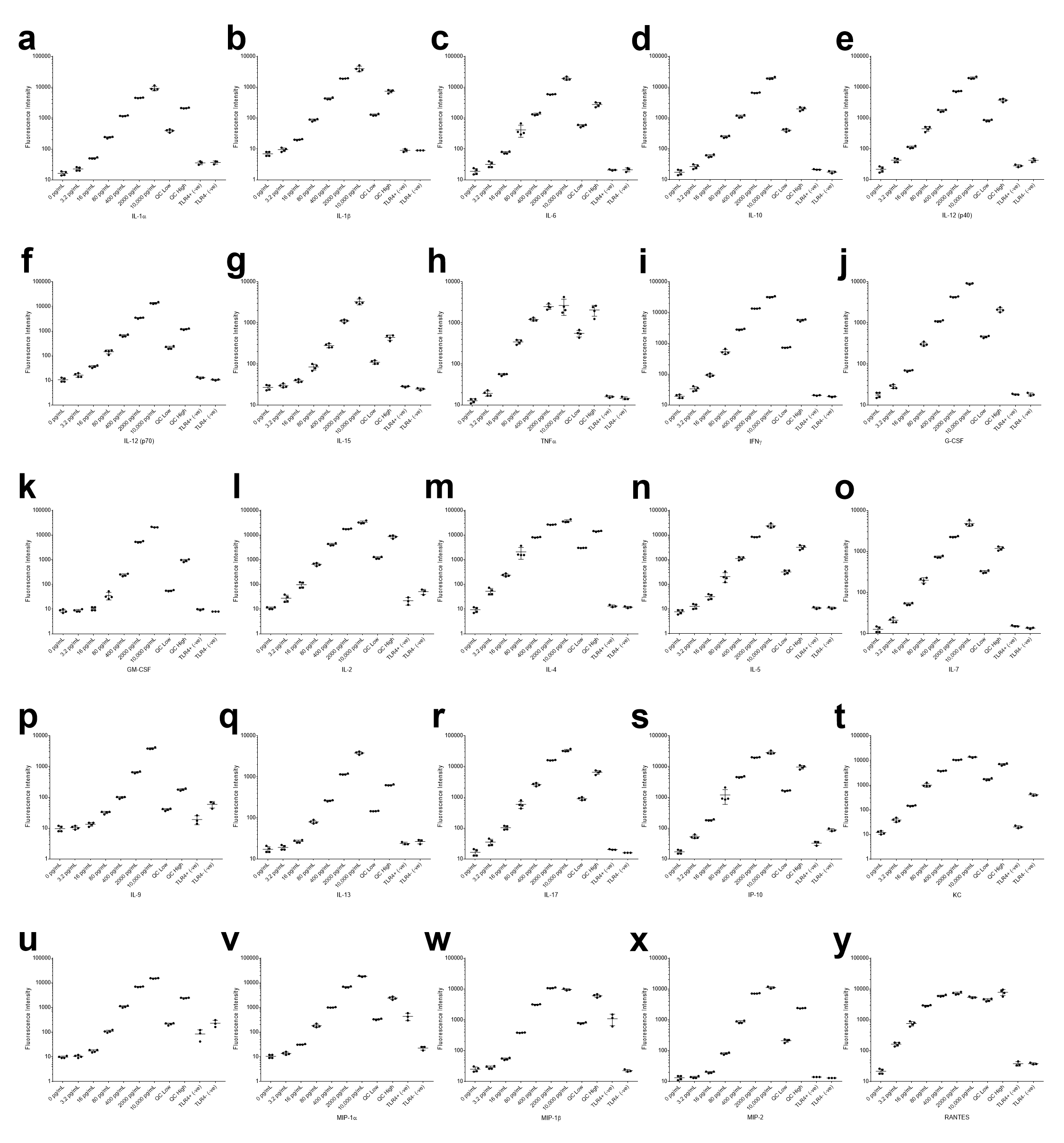

Supplement: S9 Fig — (a-y) Results of four independent replicates of manufacturer prepared standards were plotted as fluorescence intensity for each individual cytokine, with error bars indicating the standard deviation of the mean for each condition. In the case of G-CSF, GM-CSF, KC, RANTES, MIP-2, MIP-1B, and MIP1a, one of the values for 10,000 pg/mL presented as an outlier and was eliminated using Grubb’s Outlier test, with an alpha = 0.05. QC High and QC Low were high and low quality controls of unknown concentration, which were provided by the manufacturer. The negative cell controls were the basal cytokine expression of the cells in normal growth conditions with no LPS. Y-axis is plotted on a logarithmic scale to allow for viewing a large range of values on a single plot. (TIF) [file pone.0198531.s009.tif]

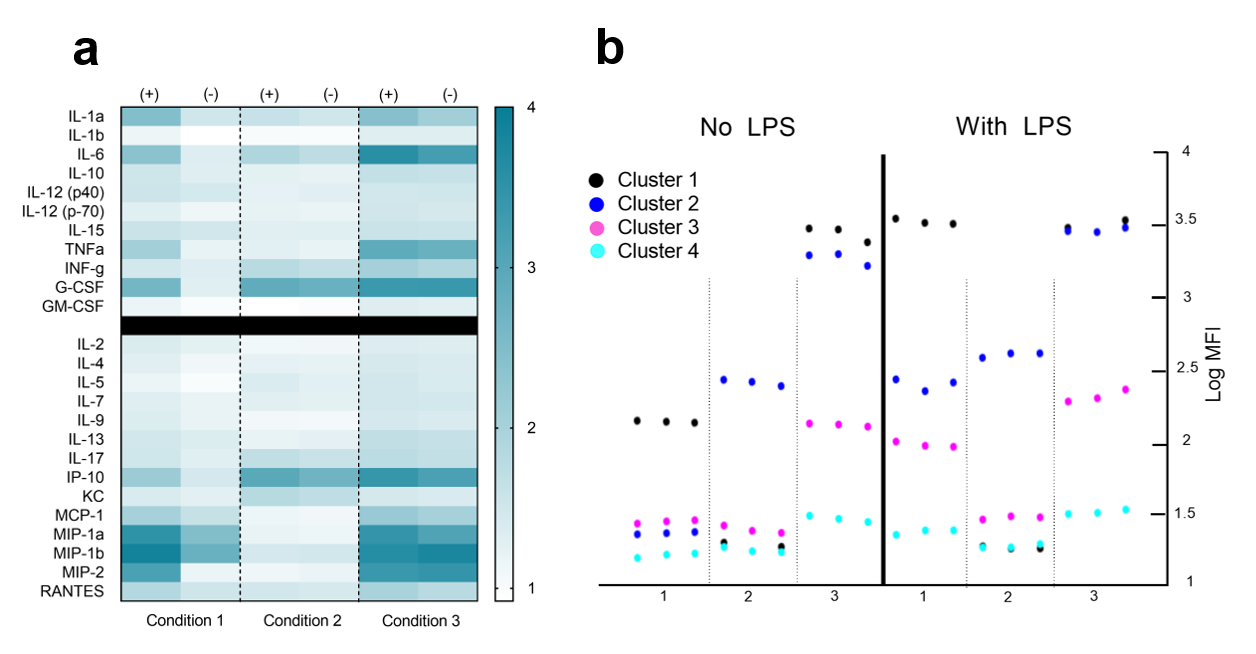

Supplement: S10 Fig — (a) Heat-map intensity of cytokine expression in conditions 1, 2, and 3, both with (+) and without (-) LPS stimulation. LPS condition is labeled on the upper axis, while condition is labeled on the lower axis. Scale bar indicates that 1 is the lowest and 4 is the highest intensity. Values are plotted as the mean log MFI, n = 3. (b) K-means clustering of cytokine expression in all conditions, with and without LPS stimulation. Cluster 1 (Black) = MIP-1α, MIP-1β, and MIP-2. Cluster 2 (Blue) = IL-6, IP-10, and G CSF. Cluster 3 (Magenta) = IL-1α, MCP-1, TNFα, INFγ, and RANTES. Cluster 4 (Cyan) = GM CSF, IL-1β, IL-2, IL-4, IL-5, IL-7, IL-9, IL-10, IL-12 (p40), IL-12 (p70), IL-13, IL-15, IL-17, and KC. (TIF) [file pone.0198531.s010.tif]

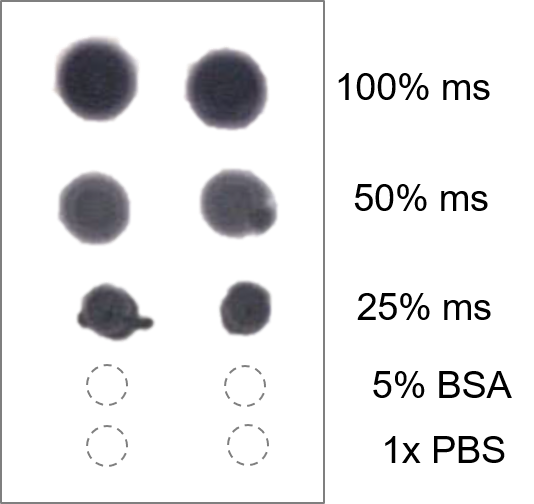

Supplement: S11 Fig — Dark spots indicate a positive result for all dilution of mouse serum and negative controls (5% BSA and 1x PBS) have no spots. (TIF) [file pone.0198531.s011.tif]
